# Thermodynamic effects of solid electrolyte interphase formation from solvation and ionic association in water-in-salt electrolytes

**DOI:** 10.1039/d6fd00035e

**Published:** 2026-08-24

**Authors:** Daniel M. Markiewitz, Michael McEldrew, Conor M. E. Phelan, Qianlu Zheng, Jasper Singh, Robert S. Weatherup, Rosa M. Espinosa-Marzal, Martin Z. Bazant, Zachary A. H. Goodwin

**Affiliations:** a Department of Chemical Engineering, Massachusetts Institute of Technology Cambridge Massachusetts 02139 USA Bazant@mit.edu; b Department of Materials, University of Oxford Parks Road Oxford OX1 3PH UK zac.goodwin@materials.ox.ac.uk; c Department of Civil and Environmental Engineering, The Grainger College of Engineering, University of Illinois Urbana-Champaign Urbana Illinois 61801 USA; d Diamond Light Source Didcot Oxfordshire OX11 0DE UK; e The Faraday Institution, Quad One, Harwell Science and Innovation Campus Didcot OX11 0RA UK; f Department of Materials Science and Engineering, The Grainger College of Engineering, University of Illinois Urbana-Champaign Urbana Illinois 61801 USA; g Department of Mathematics, Massachusetts Institute of Technology Cambridge Massachusetts 02139 USA; h John A. Paulson School of Engineering and Applied Sciences, Harvard University Cambridge Massachusetts 02138 USA

## Abstract

Water-in-salt electrolytes (WiSEs) are a promising class of next-generation electrolytes. Unlike classical dilute electrolytes or more conventional battery electrolytes, WiSEs are characterized by their super-concentrated salt content and low water content, which give rise to an expanded electrochemical stability window (ESW). The expansion of the ESW is due in part to the formation of an inorganic solid electrolyte interphase (SEI) that passivates the anode; this principle also proves important in commercial Li-ion batteries, where graphite and Li-metal anodes operate at potentials outside the ESW of conventional carbonate electrolytes, as well as extending beyond Li-ion technologies. Solvation structure and ionic association are key descriptors for understanding the expansion of the ESW. In particular, because the reactions that lead to SEI formation, or to cathode electrolyte interphase (CEI) formation, occur at the electrode–electrolyte interface, the distribution of reactants and their solvation environments is critical. In the absence of reactions, this interfacial distribution is referred to as the electrical double layer (EDL). Here, we further develop and analyze a recently proposed thermodynamic theory of hydration and ionic association in the EDL of WiSEs. We parameterize the theory using bulk molecular dynamics simulations and benchmark it against EDL simulations, finding good qualitative agreement. Using this thermodynamic framework, we rationalize an expansion in the ESW through changes in bulk electrolyte activity, via the Nernst equation, which directly alters electrolyte stability, and through thermodynamic effects on reaction kinetics, *via* the concentration of reactant species in the Helmholtz layer. Overall, this formalism directly links solvation and ionic aggregation to changes in reactivity, providing key insights into the thermodynamic factors that influence SEI formation in WiSEs. The framework can also be applied to other liquid electrolytes, including conventional carbonate electrolytes, solvent-in-salt systems, and Na-ion electrolytes, to understand how solvation and ionic association influence ESW expansion and SEI formation.

## Abstract 2

Water-in-salt electrolytes (WiSEs) are a promising class of next-generation electrolytes.

## Graphical abstract

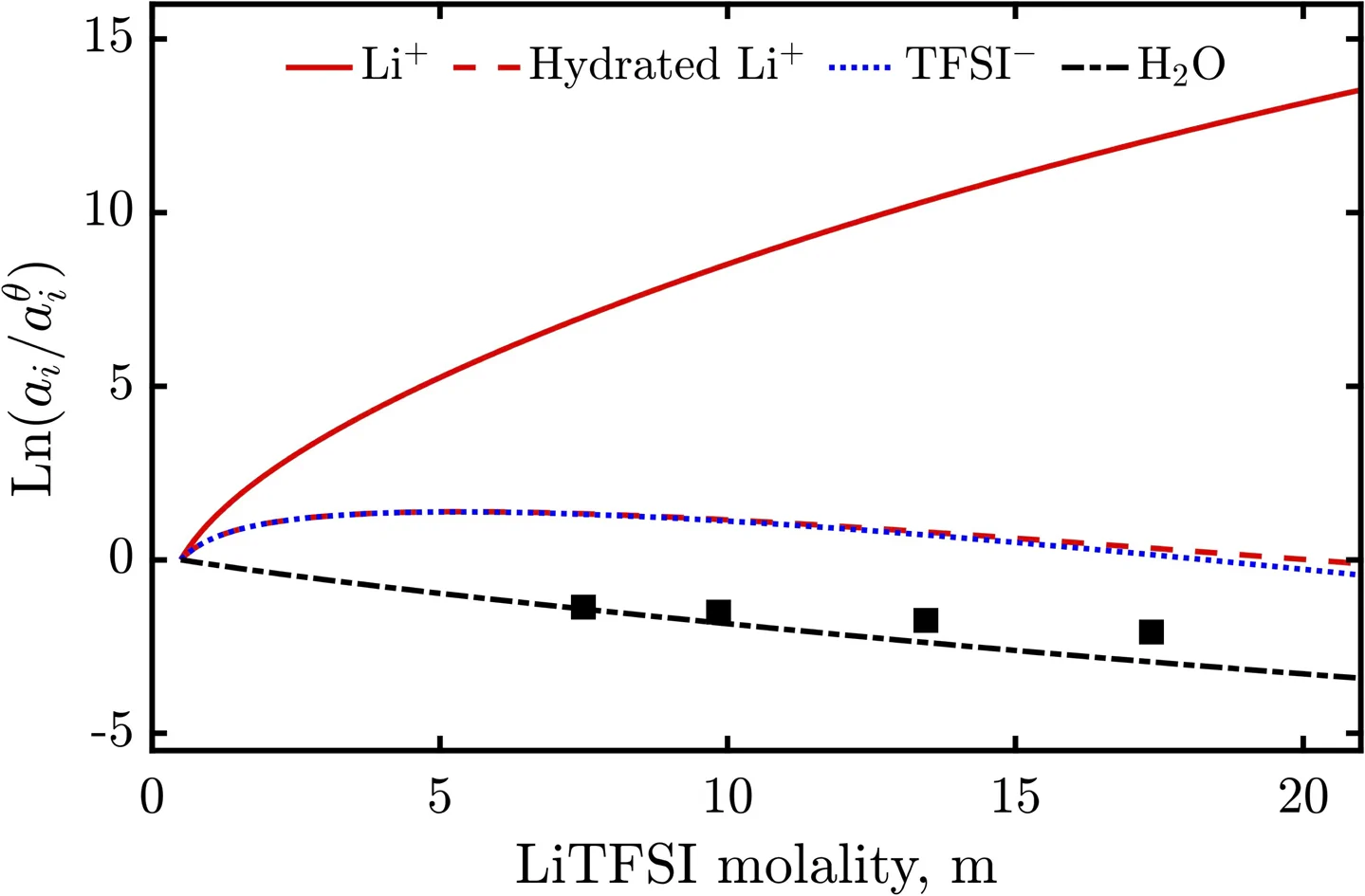

## 1. Introduction

Lithium-ion batteries (LIBs) have transformed many technologies over the past decades, including portable electronic devices, transportation, and grid-level storage of renewable energy.^1–9^ A prototypical LIB electrolyte consists of a mixture of carbonate-based solvents, such as ethylene carbonate (EC) and ethyl methyl carbonate (EMC), with LiPF_6_ at concentrations of ∼1 M, often with additives to achieve the desired performance.^1,3,10^ While the cost per kWh of LIBs continues to reduce year on year, there remains a great deal of interest in the development of novel chemistries in order to improve safety, capacity, sustainability and lifetime, among other goals.^1–9^ These advances are largely being pursued by changing the electrodes of LIBs—which comprise much of the volume, weight, and cost of LIBs^11^—to Li-metal or Si anodes,^12–15^ higher-energy-density cathodes,^16^ and chemistries beyond Li-ion systems,^17–19^ for example. However, the electrolyte that transports Li^+^ ions (or other active ions) between the electrodes remains fundamentally important,^20–32^ as all components must function efficiently together. For instance, interphases form between the electrodes and the electrolyte: the solid electrolyte interphase (SEI) on the anode side and the cathode electrolyte interphase (CEI) on the cathode side. These interphases can form through the degradation of both the electrolyte and the electrode, but their formation greatly improves coulombic efficiency by suppressing continuous electrolyte decomposition, which is key to the long-term health of the battery.^1–9,15^

Understanding SEI and CEI formation is challenging because it occurs at a solid–liquid interface, where electron-transfer processes and reactions take place over length and time scales that exceed what is feasible with conventional atomistic modeling approaches.^1–9^ Machine learning interatomic potentials (MLIPs) show promise for revolutionizing this area.^33,34^ Unfortunately, these methods can be difficult to train for such electrolytes, often lack long-range electrostatics, and it can also be challenging to incorporate information about reaction barriers.^35–38^ Therefore, it is important to develop simple analytical approaches that aid our understanding of interphase formation.

It is well established in the battery community that the coordination environments of Li^+^ ions (or other active cations) correlate with coulombic efficiency, transport properties, and interphase formation, among other properties.^9,39–56^ In terms of interphase formation, the relevant reactions are often understood in terms of changes in frontier orbitals (HOMO and LUMO levels) and how these depend on the coordination structure of the electrolyte.^49,55–59^ However, computing these properties for an electrolyte is costly, and even more so when interfacial environments are considered.^59^ An alternative approach is to understand changes in stability and reactivity from thermodynamic effects, such as changes in activity. One example is the liquid Madelung potential proposed by Takenaka *et al.*,^60^ which links Li^+^ activity to the electrostatic potential in the electrolyte. Another is the thermoreversible ionic aggregation and solvation theory of McEldrew *et al.*,^61^ which directly links coordination environments to activity,^62^ among other properties.^63–65^ These changes in activity directly translate to shifts in redox potentials through the Nernst equation, thereby thermodynamically changing the stability of the electrolyte,^62^ and are linked to changes in frontier orbitals.^66^

Another important aspect of interphase formation is the electrical double layer (EDL), *i.e.*, the ion distribution near the charged interface.^67–69^ This is important because the species that react to form the SEI or CEI must be present at the interface for electron-transfer reactions to occur, with reaction rates proportional to the concentration of the reactants.^70^ Because simulating the EDL of electrolytes is costly and challenging, there is strong motivation to develop simple theoretical approaches. Many theories for predicting the EDL structure exist, ranging from simple local-density approximations^67–69^ to more sophisticated approaches.^71^ In LIBs, specific interactions between ions and solvent are important, and an approach that can capture these interactions is essential.^65^ As far as we are aware, the only theory that can consistently predict coordination environments in the EDL is the work of Goodwin, Markiewitz, and co-workers,^65,72–75^ which extends the earlier work of McEldrew *et al.*^61–64^ This approach has been investigated for several electrolytes, including ionic liquids (ILs),^63^ salt-in-ILs,^64,73^ water-in-salt electrolytes (WiSEs),^62,74^ and conventional battery electrolytes.^75,76^

WiSEs are a promising class of electrolytes for battery applications.^22,77–82^ They offer improved safety because of their water-based solvent, while the high salt concentration (*e.g.*, 21 m LiTFSI) can extend the electrochemical stability window (ESW) to ∼4 V, in contrast to dilute aqueous electrolytes.^41,62,81,83–85^ Moreover, ionic association and Li^+^ hydration are known to be important in 21 m WiSEs, as percolating ionic networks with interpenetrating water domains have been found, while still allowing facile Li^+^ transport.^42,62,86–93^ McEldrew, Markiewitz, and co-workers^62,74,84^ have previously investigated bulk stability and EDL properties at lower concentrations (up to 15 m), gaining insight into the role of ionic association and hydration in determining activity and EDL structure in WiSEs. However, the EDL of 21 m WiSEs has not previously been investigated using this approach,^74^ despite being the most promising regime for achieving the expanded ESWs for high-energy density batteries.

In this paper, we investigate the bulk and EDL properties of 21 m LiTFSI WiSEs using molecular dynamics (MD) simulations and theory, with the aim of gaining insight into the thermodynamic factors that influence electrolyte stability and interphase formation. We first outline our theory of hydration and ionic association in LiTFSI WiSEs and describe how we have extended the analysis to investigate EDL properties at 21 m concentration. We then validate the theory against atomistic MD simulations of the EDL in 21 m LiTFSI and find good qualitative agreement. Having further validated the theory, we discuss the implications of its thermodynamic predictions for solid electrolyte interphase formation, beginning with bulk stability changes as a function of concentration and then turning to thermodynamic effects on reaction kinetics under specific conditions. Finally, we describe how this formalism can be generalized to other electrolytes of interest for Li-metal anodes and related systems, and we discuss how the role of the electrode should be incorporated more fully into the model.

## 2. Theory

Here, we present an overview of the EDL theory of WiSEs with thermoreversible associations, since it is important to understand the details of the predicted changes in thermodynamic stability of the electrolyte, and its implications for SEI formation. In ref. 61–66 and 72–76, the theory is outlined in more detail for WiSEs, and other electrolytes, in the bulk and at electrified interfaces, if further information is required.

The WiSE system studied here comprises cations (+), anions (−), and water (0). These species are assumed to exist in an incompressible lattice-gas.^62^ We define the volume of a lattice site to be that of a water molecule, *v*_0_. The volumes of the other species are expressed relative to *v*_0_ through the number of lattice sites they occupy, *ξ*_*j*_ = *v*_*j*_/*v*_0_.

To treat correlations beyond mean-field, we consider the formation of associations between cations and anions, and between cations and water.^61,62^ We do not consider associations between anions and water, and between water molecules, as these have been shown to be smaller effects.^62^ Cations form a maximum of *f*_+_ associations, anions form a maximum of *f*_−_ associations, and water forms a maximum of 1 association; referred to as the functionality of a species.^61,62^ Since *f*_±_ > 1 for WiSEs, a polydisperse set of Cayley tree clusters form, which can be uniquely classified by the rank *lms*, *i.e.*, the number of cations *l*, anions *m*, and water *s* in the cluster.^61,62^ The Cayley tree assumption is necessary to keep this theory analytically tractable and physically intuitive. This assumption has been shown to work well for WiSE, and a variety of other concentrated electrolytes.^62–64,66,76,94^ When *f*_±_≥ 2, which is typical for WiSEs, a percolating ionic network (also referred to as a gel) can form.^61^

One of the central variables of our theory is the volume fraction of each species, *ϕ*_*j*_, where *j* is +, −, 0, or the dimensionless concentrations of species *c*_*j*_ = *ϕ*_*j*_/*ξ*_*j*_. A quantity we aim to find from our theory is the dimensionless concentrations of each cluster rank, *c*_*lms*_. When a gel phase exists we split up the total volume fractions into sol and gel contributions, *ϕ*_*j*_ = *ϕ*^sol^_*j*_ + *ϕ*^gel^_*j*_, where these volume fractions are determined from Flory’s post-gel convention. Finally, the dimensionless concentration of each species can be written as 
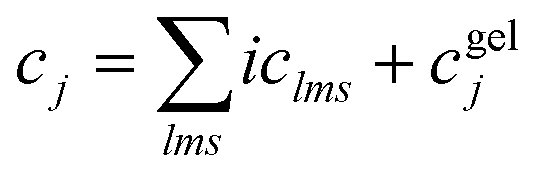
, where *i* is *l*, *m*, *s* for *j* is +, −, 0, respectively.

Following ref. 74, the free energy functional 
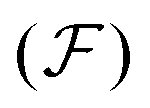
 is1
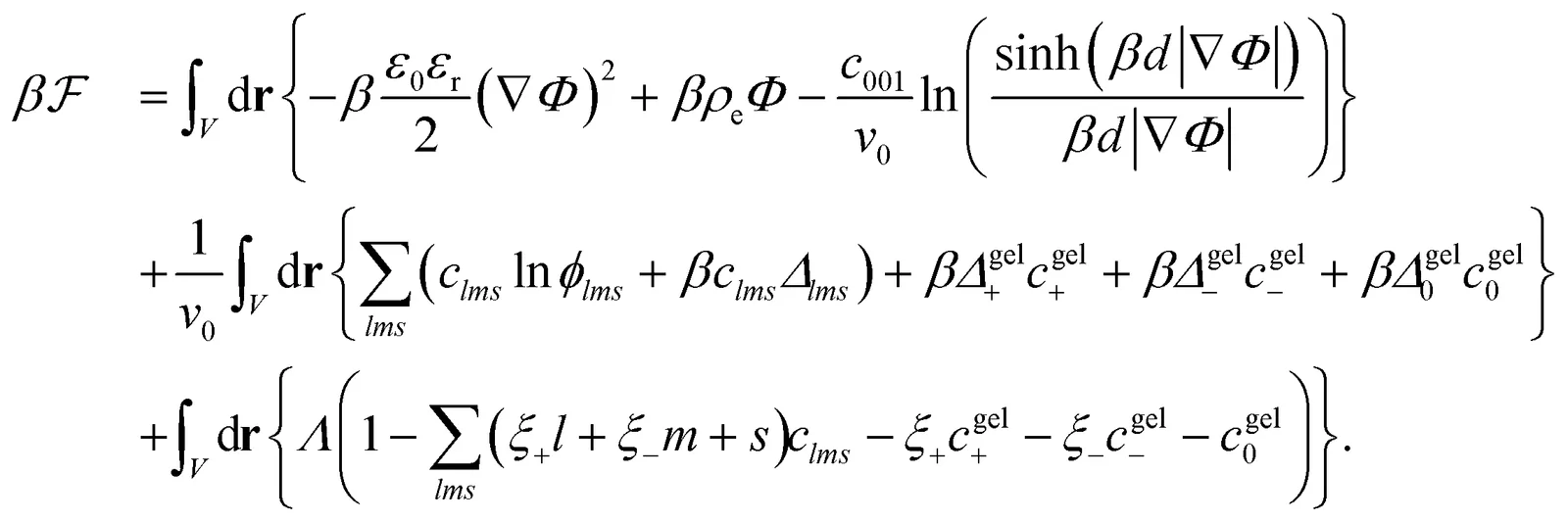
Here, *β* = 1/*k*_B_*T* is the inverse thermal energy. The first three terms (first line of the functional) represent the electrostatic contribution to the free energy, where *ε*_0_ and *ε*_*r*_ are, respectively, the vacuum and relative permittivity, *Φ*(**r**) is the electrostatic potential, *ρ*_e_(**r**) = *e*(*c*_+_ − *c*_−_)/*v*_0_ is the charge density with *e* being elementary charge, and *c*_001_ and *d* are, respectively, the dimensionless concentration of free water and its dipole moment, which is assumed to behave as a fluctuating Langevin dipole.^74,84^ The fourth term (first on the second line) is the ideal entropy from the clusters. The fifth term (second on the second line) is the free energy of forming clusters, where *Δ*_*lms*_ is the free energy of forming a cluster of rank *lms*. The sixth, seventh, and eighth terms (remaining terms on the second line) represent the free energy of species associating with the gel, *Δ*^gel^_*j*_. The final term (third line) is the Lagrange multiplier, *Λ*(**r**), which is used to enforce incompressibility in the EDL.^73,74^

We consider *Δ*_*lms*_ to have three contributions:

2 *Δ*_*lms*_ = *Δ*^comb^_*lms*_ + *Δ*^bind^_*lms*_ + *Δ*^conf^_*lms*_,

where *Δ*^comb^_*lms*_ is the combinatorial entropy, *Δ*^bind^_*lms*_ is the binding energy, and *Δ*^conf^_*lms*_ is the configurational entropy.

The combinatorial entropy for the WiSEs Cayley tree clusters^62,74^ is given by

3 *Δ*^comb^_*lms*_ = −*k*_B_*T* ln{*f*_+_^*l*^*f*_−_^*m*^*W*_*lms*_},

where4
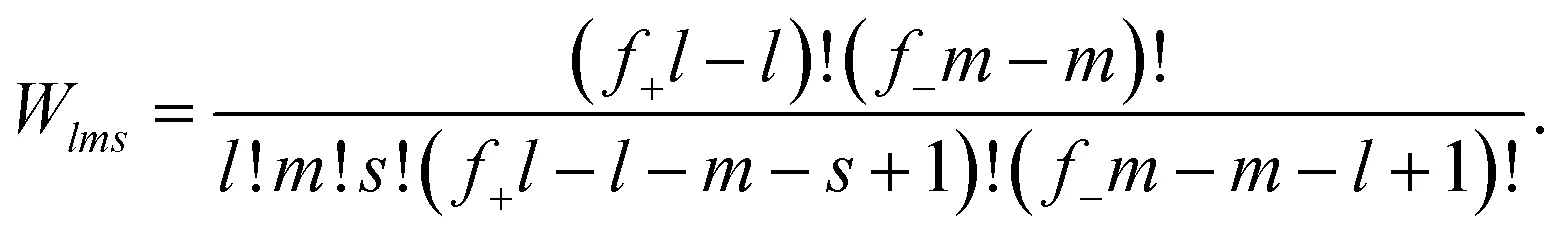


The binding energy for an *lms* cluster with *l* > 0 is

5 *Δ*^bind^_*lms*_ = (*l* + *m* − 1)Δ*u*_+−_ + *s*Δ*u*_+0_,

where Δ*u*_+*i*_ is the energy of an association between a cation and anion or water.^62,74^ When *l* = 0, the binding energy is zero.

Lastly, the configurational entropy is assumed to take the form6
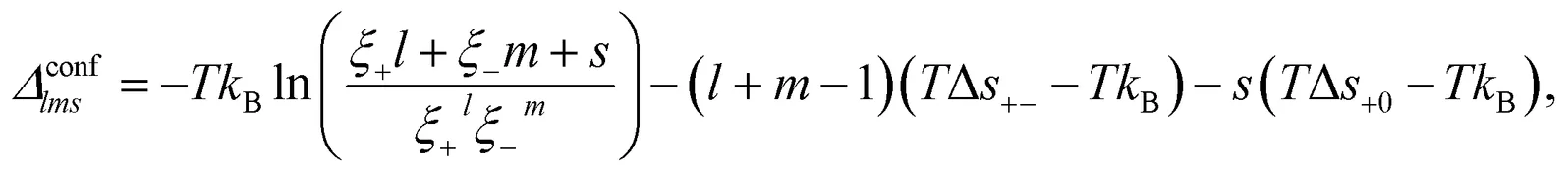
where Δ*s*_+*i*_ is the entropy of an association.^62,66^

We can calculate the electrochemical potential of the clusters through taking the derivative of the free energy with respect to *c*_*lms*_7
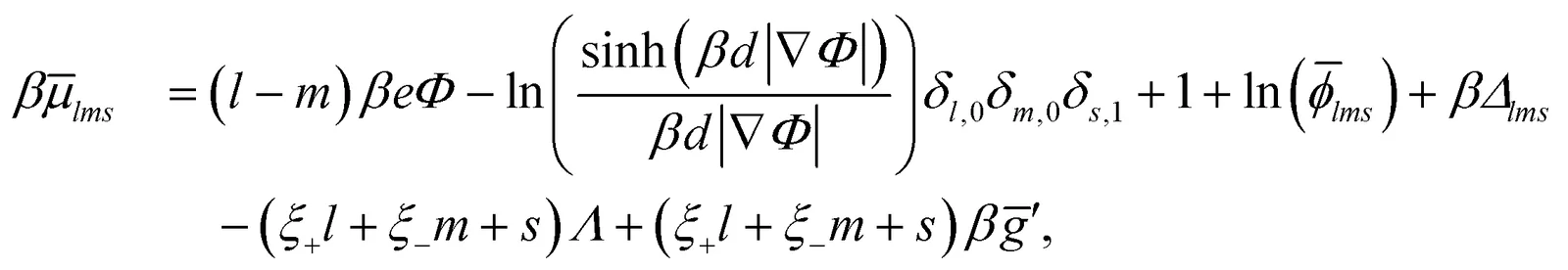
where *ḡ*′ = *c̄*^gel^_+_∂*Δ̄*^gel^_+_ + *c̄*^gel^_−_∂*Δ̄*^gel^_−_ + *c̄*^gel^_0_∂*Δ̄*^gel^_0_, with the partial derivative being with respect to *φ̄*_*lms*_. An overbar indicates that the variable is the EDL version and *Φ* is non-zero, and the absence of it indicates bulk electrolyte, *i.e.*, with zero *Φ*.

In the bulk, chemical equilibrium can be established between free species and aggregates:

8 *lµ*_100_ + *mµ*_010_ + *sµ*_001_ = *µ*_*lms*_,

which can be used to find the cluster distribution9
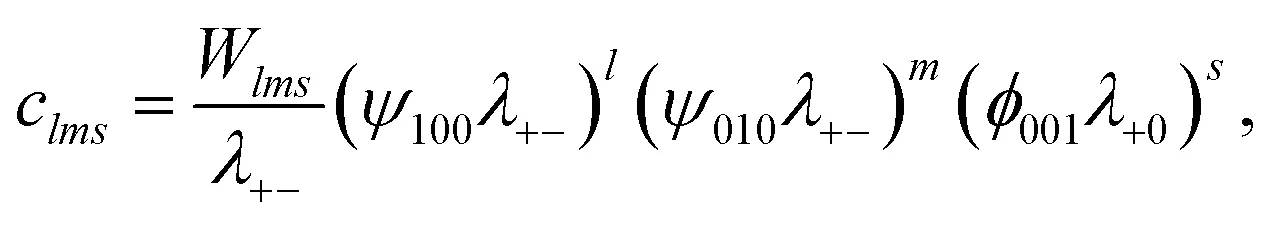
where *ψ*_100_ = *f*_+_*ϕ*_100_/*ξ*_+_ and *ψ*_010_ = *f*_−_*ϕ*_010_/*ξ*_−_ are dimensionless concentrations of free lattice sites, and *λ*_+−_ is the cation–anion association constant and *λ*_+0_ is the cation–water association constant, given by *λ*_+*i*_ = exp{−*β*Δ*f*_+*i*_}, where Δ*f*_+*i*_ = Δ*u*_+*i*_ − *T*Δ*s*_+*i*_ is the free energy of the association.

In order to compute the cluster distribution, we need to know the volume fractions of the free species. These, however, are not *a priori* known, and in fact, we wanted to find them from the theory instead of having them as an input. To overcome this obstacle, we introduce association probabilities, *p*_*ij*_, of *i* binding to *j*.^61,62^ In an independent association approximation, we can determine the bare species volume fractions as *ϕ*_100_ = *ϕ*_+_(1 − *p*_+−_ − *p*_+0_)^*f*_+_^, *ϕ*_010_ = *ϕ*_−_(1 − *p*_−+_)^*f*_−_^, and *ϕ*_001_ = *ϕ*_0_(1 − *p*_0+_).

While we seem to have simply moved the issue, as we now need to determine the association probabilities that are also not *a priori* known, we can introduce the conservation of associations and the mass action laws of the open and occupied association sites^61,62^ to determine these unknowns. The conservation of cation–anion associations is

10 *ψ*_+_*p*_+−_ = *ψ*_−_*p*_−+_ = *ζ*,

where *ψ*_+_ = *f*_+_*ϕ*_+_/*ξ*_+_ and *ψ*_−_ = *f*_−_*ϕ*_−_/*ξ*_−_ are, respectively, the number of cation and anion association sites per lattice site, and *ζ* represents the number of cation–anion associations per lattice site.^61,62^ Similarly, the conservation of cation–water association is

11 *ψ*_+_*p*_+0_ = *ϕ*_0_*p*_0+_ = *Γ*,

where *Γ* represents the number of cation–water associations per lattice site.^61,62^ The mass action law of cation–anion associations is12
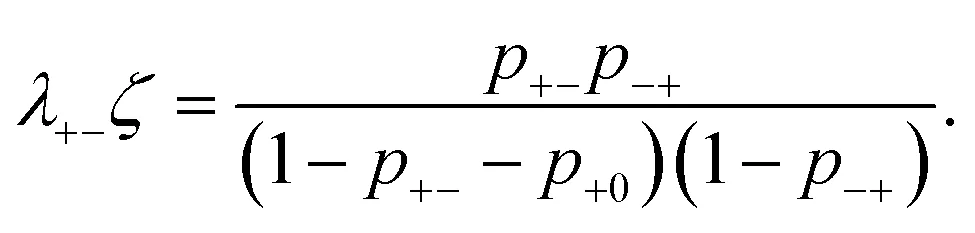
For cation–water associations it is13
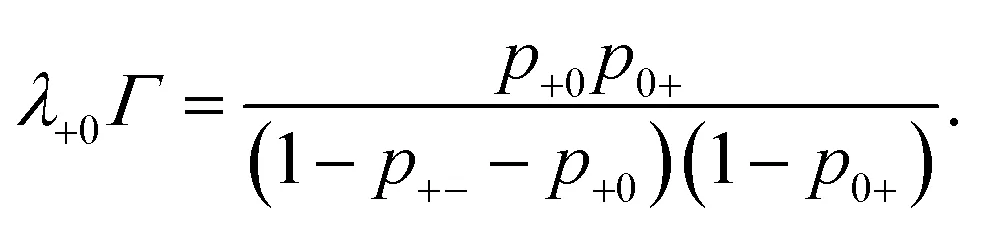
Therefore, to determine the association probabilities, all we need to know is the association constants, which is another central variable of our theory.

Since the solvation shell of Li^+^ is often full or close to being full, *i.e.*, the number of coordinating species is approximately *f*_+_, the mass action laws become challenging to solve because *p*_+−_ + *p*_+0_ ≈ 1 is near a singularity.^62^ To overcome the singularity and facilitate a simplified solution, we can introduce the so-called “sticky-cation” approximation, where we assume *p*_+−_ + *p*_+0_ = 1.^62^ The created singularities in the mass action laws, eqn (12) and (13), can be regularized by taking the ratio of these equations,14
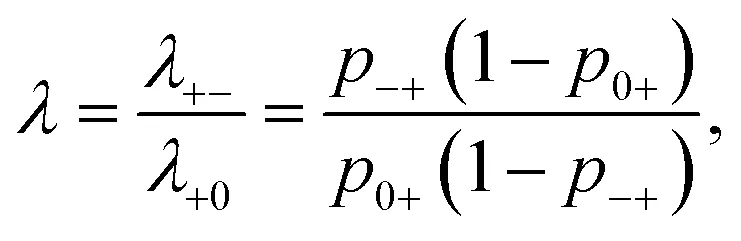
where *λ* is the cation association constant ratio. Moreover, as the sticky-cation approximation requires *s* = *f*_+_*l* − *l* − *m* + 1, the sticky-cation cluster distribution simplifies to15
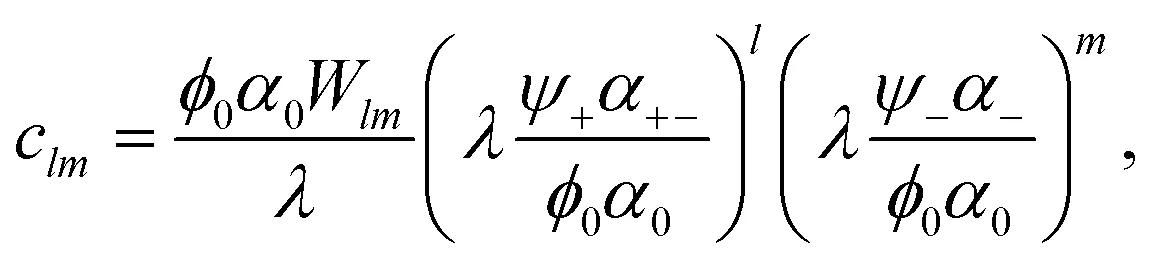
where16
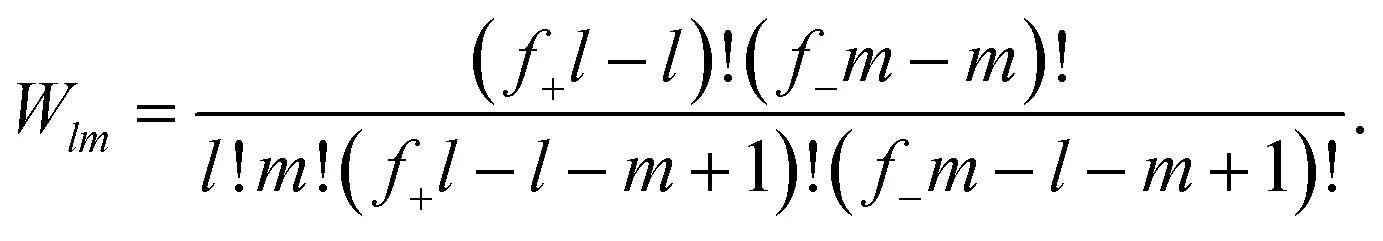
Here, *α*_0_ = 1 − *p*_0+_, *α*_+−_ = (1−*p*_+−_)^*f*_+_^ and *α*_−_ = (1−*p*_−+_)^*f*_+_^ are the fraction of free water molecules, fully hydrated cations, and free anions, respectively.

Similarly, establishing the equilibrium between the free species and the clusters *within* the EDL^65^

17 *lμ̄*_100_ + *mμ̄*_010_ + *sμ̄*_001_ = *μ̄*_*lms*_,

we obtain an analogous solution for the EDL cluster distribution^65,74^18
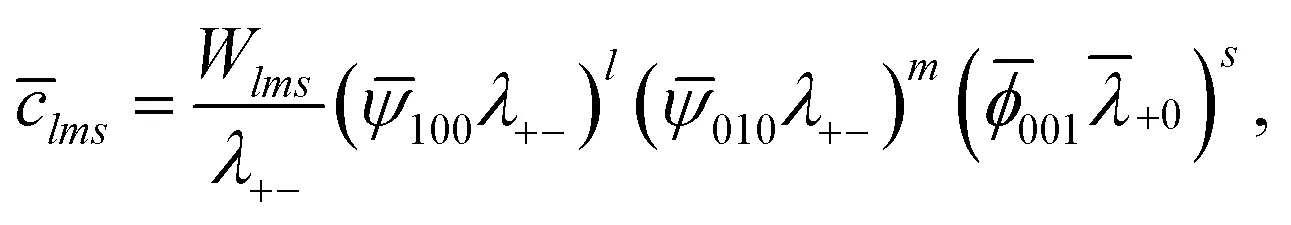
where19
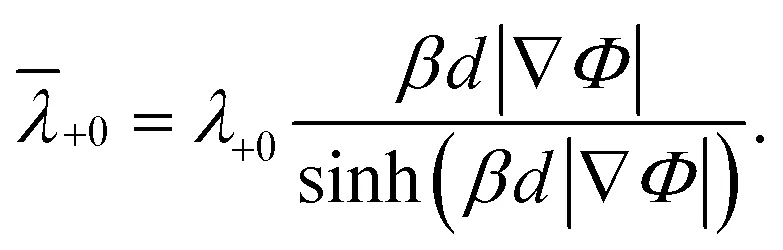
As shown above, the cation–water association constant explicitly depends on the electric field, since the energy of free water changes in the field, but bound water is assumed not to respond to the field.^74^ Analogously, we can enforce the sticky-cation approximation in the EDL with *p̄*_+−_ + *p̄*_+0_ = 1, where an equivalent set of conservation of associations and mass action laws apply, and we find an analogous sticky-cation cluster distribution, *c̄*_lm_, where *λ̄* explicitly depends on the electric field because of *λ̄*_+0_.

Finally to establish an equilibrium between the bulk and the EDL, we set the electrochemical potentials of the free species equal to each other, *e.g.*, *μ̄*_010_ = *µ*_010_.^65^ Following the work of Markiewitz and Goodwin *et al.*,^65,72–75^ these can be expressed as so-called Boltzmann closure relations to consistently establish all chemical equilibria. For the anions, we have

20 *φ̄*_010_ = *ϕ*_010_exp(*βeαΦ* + *ξ*_−_*Λ*),

and for the free water21
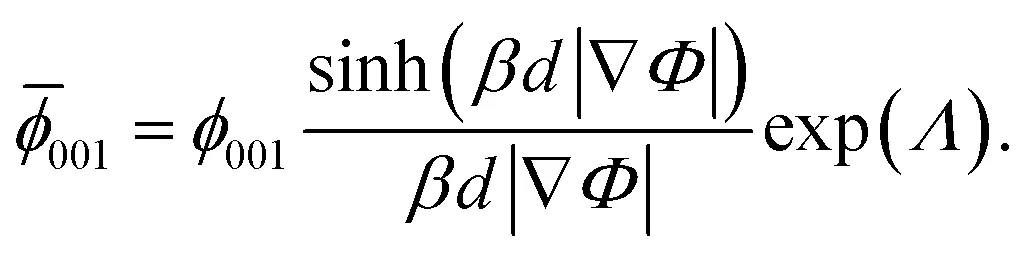
Since we will use the sticky-cation approximation, we consider the equilibrium between the fully hydrated cation in the bulk and the EDL:

22 *φ̄*_10*f*_+__ = *ϕ*_10f+_ exp(−*αβeΦ* + (*ξ*_+_ + *f*_+_)*Λ*),

where *ϕ*_10*f*+_ = (1 + *f*_+_/*ξ*_+_)*ϕ*_+_(1 − *p*_+−_)^*f*_+_^. There is, however, freedom in how we choose this closure relationship. For example, we could establish the equilibrium between free cations as

23 *φ̄*_100_ = *ϕ*_100_ exp(−*αβeΦ* + *ξ*_+_*Λ*).

While in the sticky-cation approximation, this is seemingly contradictory, since there are no free cations in the bulk or the EDL, we can regularize this problem to establish a new closure relation24
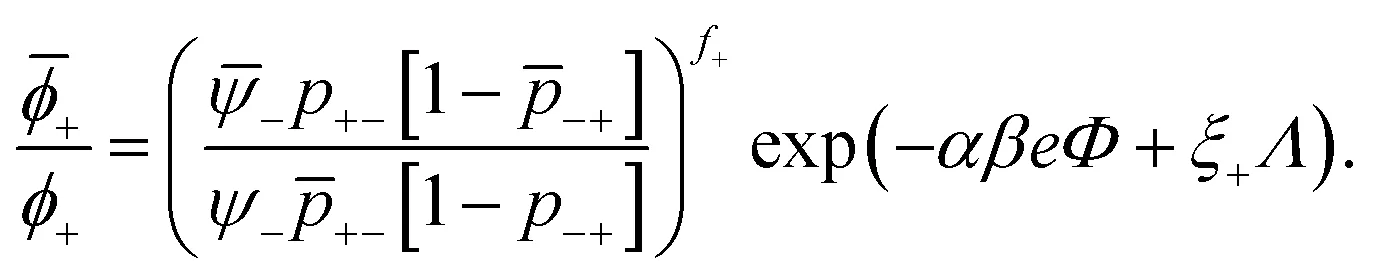


This was obtained from eqn (23) from inserting the expressions for free cations in terms of its volume fraction and association probabilities, and then using the mass action law and conservation of associations to remove the singular terms. Moreover in these closure relations, we introduce the potential rescaling parameter *α*, from ref. 69, to further include correlations beyond the mean-field ones included so far. Note that formally these closure relationships only hold in the pre-gel regime, since they are obtained from equating the pre-gel chemical potentials.^65,74^ In ref. 65, it was shown that the gel terms are small, and that we can extrapolate into the gel regime for ILs^72^ and SiILs.^73,94^ However, this extrapolation into the gel regime for WiSEs without modification has not been tested,^74^ and we test this assumption here.

To predict the EDL properties of WiSEs, we derive our modified Poisson–Boltzmann equation by taking the functional derivative of the free energy with respect to the electrostatic potential25
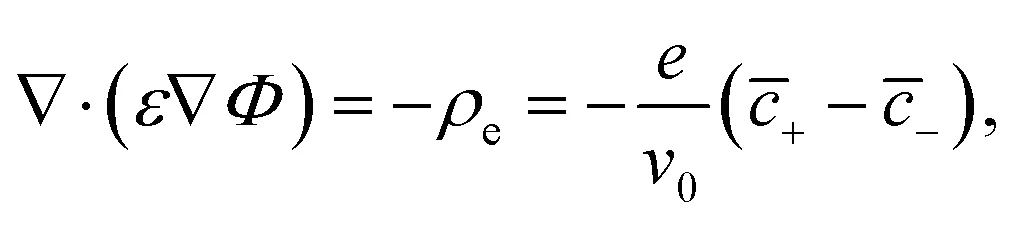
where26
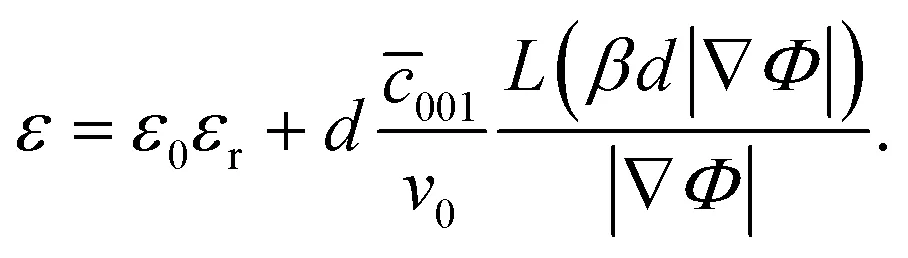
Here, *L*(*x*) = coth(*x*) − 1/*x* is the Langevin function. The procedure implemented to solve our system of equations coupled to the modified Poisson–Boltzmann equation is discussed in ref. 74. Remember, even though the gel terms are neglected in the Boltzmann closure relations, the effect of screening from the gel is still introduced through the charge density in the modified Poisson–Boltzmann equation.^65^ Some quantities will be plotted in real space relative to the Debye length, *λ*_D_, or the inverse Debye length, 
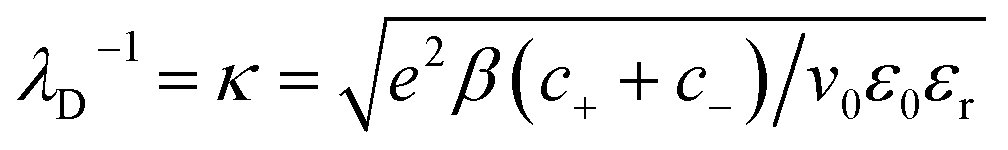
.

## 3. Methods

Here we further analyze the constant charge EDL molecular dynamics (MD) simulations from ref. 84 of 21 m LiTFSI WiSE. Therefore, we refer the reader to ref. 84 for all the details of those simulations. To compute associations in the MD simulations, we used a real-space cut-off of 2.7 Å between Li^+^ and O in TFSI^−^ and H_2_O.^74,84^ For TFSI^−^, we count the associations based on unique anions, so bidentate associations only count as a single association in this model. To calculate the aggregates, we construct an association adjacency matrix. In the EDL, we refer the reader to ref. 74 for a detailed discussion of how associations are distributed in real space.

In this work for the volume fractions, we use the same values found in ref. 62, *ξ*_+_ = 0.4 and *ξ*_−_ = 10.8. Moreover, we pick *f*_+_ = 4 and *f*_−_ = 3.^62^ Using these functionalities and the coordination numbers, we can compute the association probabilities through27
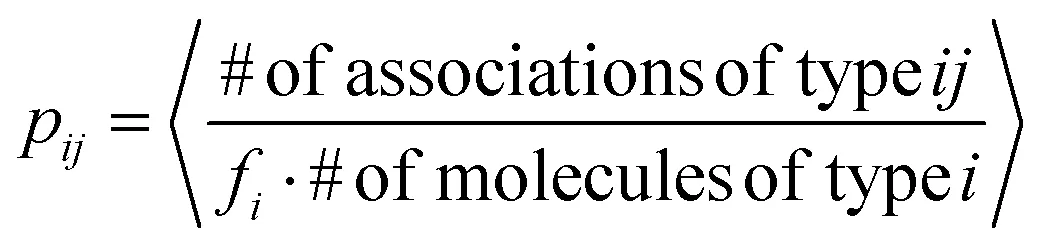
and use the mass action laws to determine the association constants, which then determines all the parameters for our theory. From our analysis of the 21 m water-in-LiTFSI system, we found *λ* = 0.2527 and *v*_0_ = 21.744 Å^3^. In addition, we used the following parameters *ε*_r_ = 10.1, and *d* = 4.995 debye.^84^

## 4. Results

In this section, we validate our proposed EDL theory for 21 m LiTFSI WiSE^62,74^ at negative and positive electrodes against atomistic MD simulations,^84^ which has not been achieved before. Notably, this is because 21 m is in the gel regime,^62,74^ and the system of equations becomes challenging to solve. Previously, in ref. 74, the polynomial formulation was investigated. This worked well for the 12 m and 15 m LiTFSI, which is why we used this method. However, it was challenging to solve this system of equations for 21 m LiTFSI, which is why this comparison was not shown.

From further testing different approaches for the 21 m LiTFSI, we found that, overall, the Boltzmann closure relations are slightly more numerically well-behaved at higher concentrations. As such, we investigated alternative forms of the closure relationship, such as the new one outlined in the Theory section. However, we found that the hydrated-cation Boltzmann closure was more robust than other approaches, as eqn (24). Therefore, we stick to this formulation here. Note that these Boltzmann closure relations are formally derived in the pre-gel regime, and additional terms from the gel could be included, but it has previously been found that these have a small effect.^65^ To further help with numerical solutions and comparisons with experiments, we introduced a consistent charge rescaling parameter,^69^ which makes the agreement more qualitative than in ref. 74 for the 12 m and 15 m LiTFSI WiSEs. We used the largest value of *α* = 0.3 (ref. 69) that would allow us to solve the system of equations. Here, we use this value for all calculations.

### 4.1. Negative EDL

**Fig. 1:**
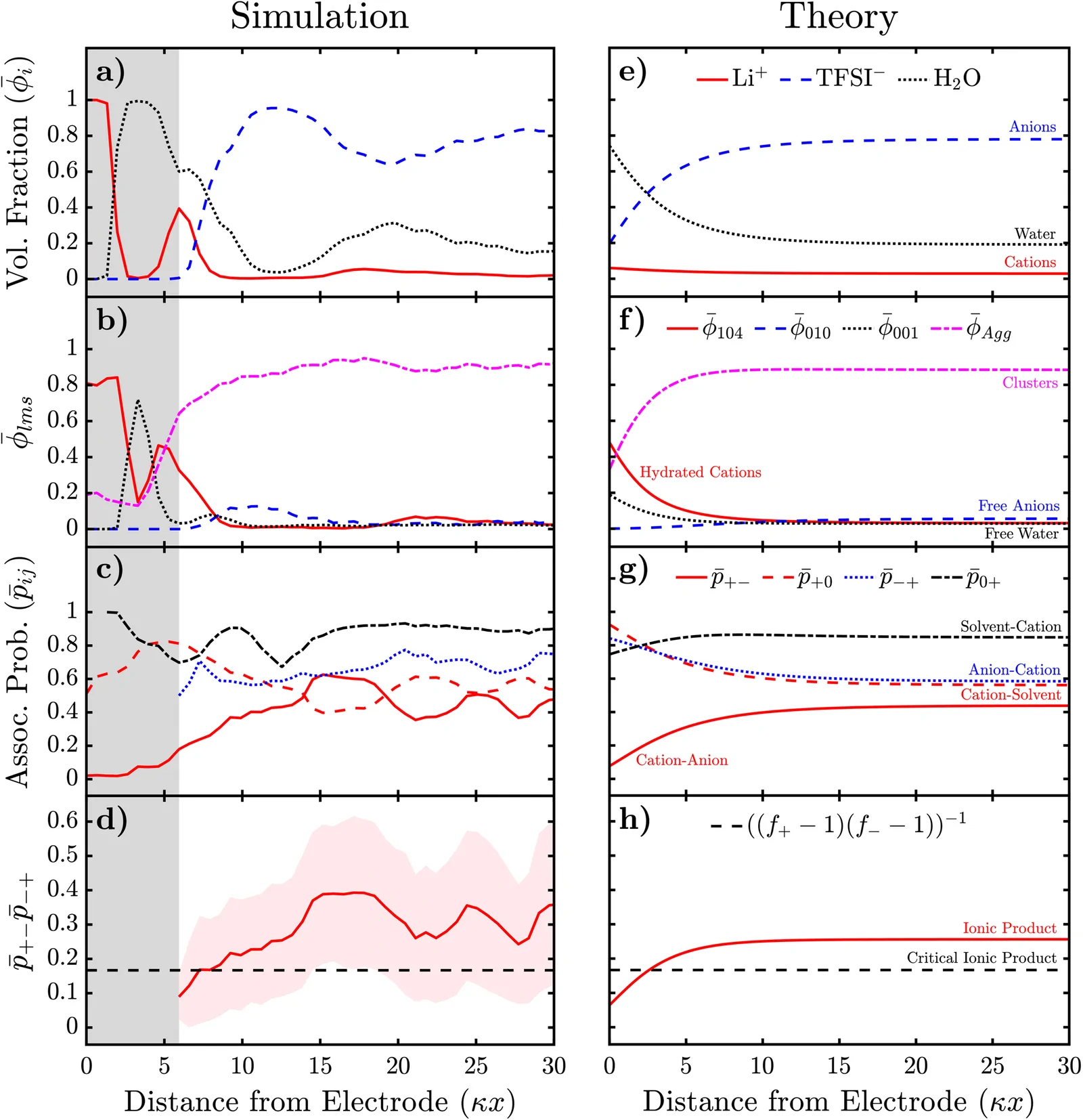
EDL of 21 m WiSEs at a negative electrode (*σ* = −0.2 C m^−2^) as a function from the interface, in dimensionless units, where *κ* is the inverse Debye length, from simulations (a–d) and theory (e–h). In the former, the grey region indicates the minimum distance from the electrode at which a species was never found. (a and e) Volume fraction of each species (*φ̄*_*i*_). (b and f) Volume fractions of hydrated cations (simulation *φ̄*_10*x*_ and theory *φ̄*_104_), free anions (*φ̄*_010_), free water (*φ̄*_001_), and aggregates and gel, if present (*φ̄*_Agg_). (c and g) Association probabilities (*p̄*_*ij*_). (d and h) Product of the ionic association probabilities, *p̄*_+−_*p̄*_−+_, where the dashed line indicates the critical line for gelation and the red shaded region is the standard deviation.

In Fig. 1, we display our comparison between theory and simulation for the anode of 21m WiSE, where the grey region in the simulations denotes where not all species can be detected in the MD simulation. This demarcates the onset of where comparison to predictions in the theory should break down and where surface effects can become important (such as blocking of association sites and interactions with the electrode).^74^ Hereon out, we refer to this region as the Helmholtz region.^75^

First, we compare the volume fractions of each species in the EDL, *φ̄*_*i*_, as seen in Fig. 1(a) and (e) for the simulation and theory, respectively. In the simulation, we find oscillations of *φ̄*_−_ in the diffuse part of the EDL, with a strong decay towards the Helmholtz region, with the anions being completely depleted in this region. On the other hand, we find *φ̄*_+_ and *φ̄*_0_ have small oscillations in the diffuse EDL, but they accumulate strongly before the Helmholtz region. Moreover, we find two distinct layers of Li^+^, one being right at the interface of the electrode in the Helmholtz region, and the other at the boundary of the Helmholtz region with the diffuse EDL, with a substantial water layer between them. The theory predictions are able to qualitatively capture these changes before the Helmholtz region, *i.e.*, the diffuse EDL, but the theory cannot capture the oscillations and layering that occur, since it is a local density approximation. Specifically, the theory predicts a monotonic decay of anions, and a monotonic increase of cations and water.

Next, we compare the volume fractions of some chosen clusters, *φ̄*_*lms*_, see Fig. 1(b) and (f), which allows us to understand the changes in species volume fractions in more detail. In the MD simulations, the volume fraction of free anions, *φ̄*_010_, remains small throughout the EDL and bulk region, indicating most anions exist in aggregates. Similarly, there is a small volume fraction of hydrated cations and water in the bulk and diffuse EDL, but there is a notable increase in them towards the interface. We find that the two layers of Li^+^ previously described are largely composed of hydrated cations, while the water layer between them is mostly free water. We also quantify the volume fraction of remaining aggregates and gel phase, calculated from 
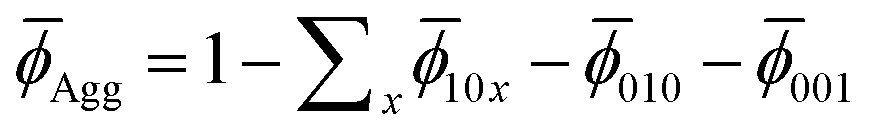
. In the bulk and diffuse EDL, the aggregates/gel are in the majority, and only strongly dissipate when in the Helmholtz region, indicating that the aggregates/gel must be contributing substantially to the screening of the electrode. Again, the theory qualitatively captures these changes, in a smooth way, up to the Helmholtz region in the MD simulations.

Finally, we discuss how the association probabilities change within the EDL, which as far as we are aware no other theory is able to predict. In Fig. 1(c) and (g) we show *p̄*_*ij*_ from simulation and theory, respectively. In the diffuse region of the simulations there are oscillations in *p̄*_+0_ (the probability of cations associating to water), which transition into a steady increase towards the Helmholtz region. In contrast, *p̄*_0+_ remains approximately constant, with a slight decrease just before the Helmholtz layer, albeit with some fluctuations. In the theory, *p̄*_+0_ steadily increases in the EDL, but *p̄*_0+_ steadily decreases. Therefore, our theory has some success in describing these changes in coordination environments.

The cation–anion association probability, *p̄*_+−_, decreases substantially in the EDL from the simulations, while the anion–cation association probability, *p̄*_−+_, remains roughly constant in the simulations. Our theory also predicts *p̄*_+−_ to decrease in the EDL, but for *p̄*_−+_ to increase to a lesser extent. Our theoretical approach describes well the cation coordination environment well, but struggles to predict the anion coordination environment.

By taking the product of these probabilities, *p̄*_+−_*p̄*_−+_, we can determine bond percolation for the Bethe lattice that the aggregates exist on. If *p̄*_+−_*p̄*_−+_ > [(*f*_+_ − 1)(*f*_−_ − 1)]^−1^, there is a percolating ionic network, *i.e.*, a gel phase. In bulk 21 m LiTFSI WiSE, the percolating ionic network is known to exist, with the gel-point being determined to be close to 18 m in LiTFSI (from the bond percolation criteria). In the simulations, as displayed in Fig. 1(d), we see in the bulk and diffuse EDL that a gel phase exists, but close to the Helmholtz region the network is destroyed as this product dips below the critical value, which corresponds well to the reduction in aggregates/gel observed in Fig. 1(b). The theory, shown in Fig. 1(h), is able to capture the changes in the percolating ionic network well, where we predict a gel phase in the bulk and diffuse EDL, but close to the interface, the criteria drops below the critical value.

Overall, in Fig. 1 we have demonstrated the changes in composition and coordination environments of 21 m LiTFSI WiSE EDL at negative electrodes, and found reasonable agreement with the theory and simulations. As previously discussed, the largest differences between these methods occur in the Helmholtz region, where not all species are present and surface effects become important. In ref. 75, it was suggested that an approach to describe the differences in the Helmholtz region was to consider the interface blocking association sites and interacting with them, manifested through an apparent reduction in the functionalities, *f*_*i*_. If we propose that *f*_+_ changes from 4 to 3 in the Helmholtz region, we might expect to observe a decrease in *p̄*_+*j*_ in the simulations in this region, as a value of 4 was used in Fig. 1, which is what we can see for *p̄*_+*j*_.

**Fig. 2:**
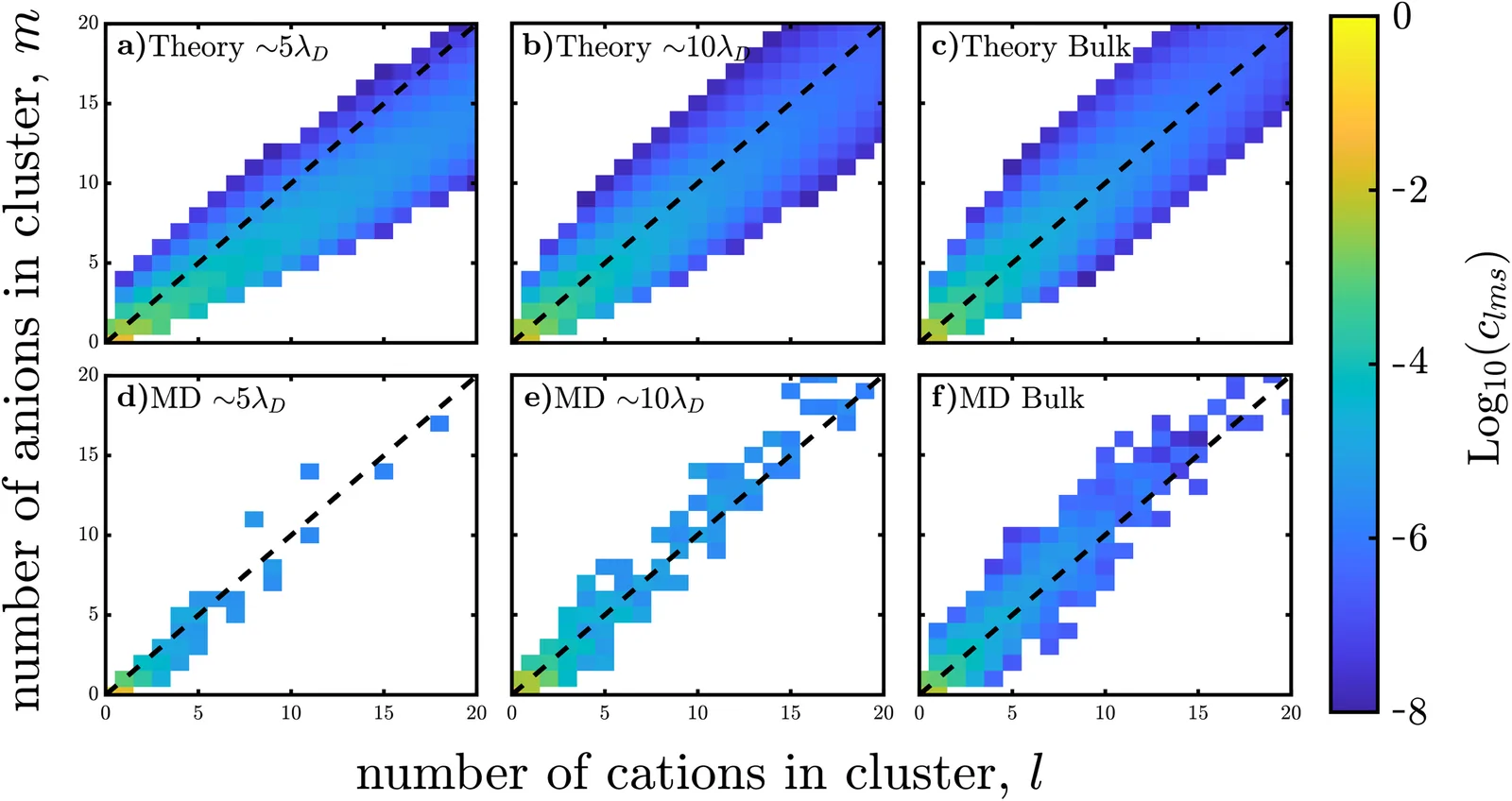
Cluster distribution of 21 m water-in-LiTFSI near the negative electrode (*σ* = −0.2 C m^−2^) at different distances from the interface, from theory (a–c) and simulations (d–f).

Our approach also allows us to investigate the cluster distribution, *c̄*_*lms*_, in different regions of the EDL, which we display in Fig. 2 from simulation and theory. Note that we use the sticky-cation approximation, so we only need to plot *c̄*_*lm*_. In the bulk, we observe the MD cluster distribution has a slightly negative bias to the aggregates, since *f*_+_ > *f*_−_, with clusters containing up to 12 cations and 12 anions, as seen in Fig. 2(f). The theory has a similar distribution, see Fig. 2(c), but with a longer tail in the distribution. In the diffuse EDL, at ∼10*λ*_D_ from the interface, we find that the MD cluster distribution has increased the concentrations of some larger clusters, consistent with the observation that the gel phase is being dismantled closer to the interface, as displayed in Fig. 2(e). For small clusters (*l* < 5), there is a positive bias of these aggregates, but for larger aggregates there is a negative bias. The theory captures the extended cluster distribution, but predicts a positive bias for all aggregates, shown in Fig. 2(b), indicating a breakdown in our assumed cluster distribution. Finally, just outside the Helmholtz region (∼5*λ*_D_), our simulations [Fig. 2(d)] show a diminished cluster distribution with positive bias, demonstrating the screening ability of these aggregates. The theory, see Fig. 2(a), has a strongly positive bias for the charge of clusters and the cluster distribution has grown relative to the diffuse EDL, which can be attributed to the theory over-predicting the stability of the gel phase 5*λ*_D_ from the interface.

### 4.2. Positive EDL

**Fig. 3:**
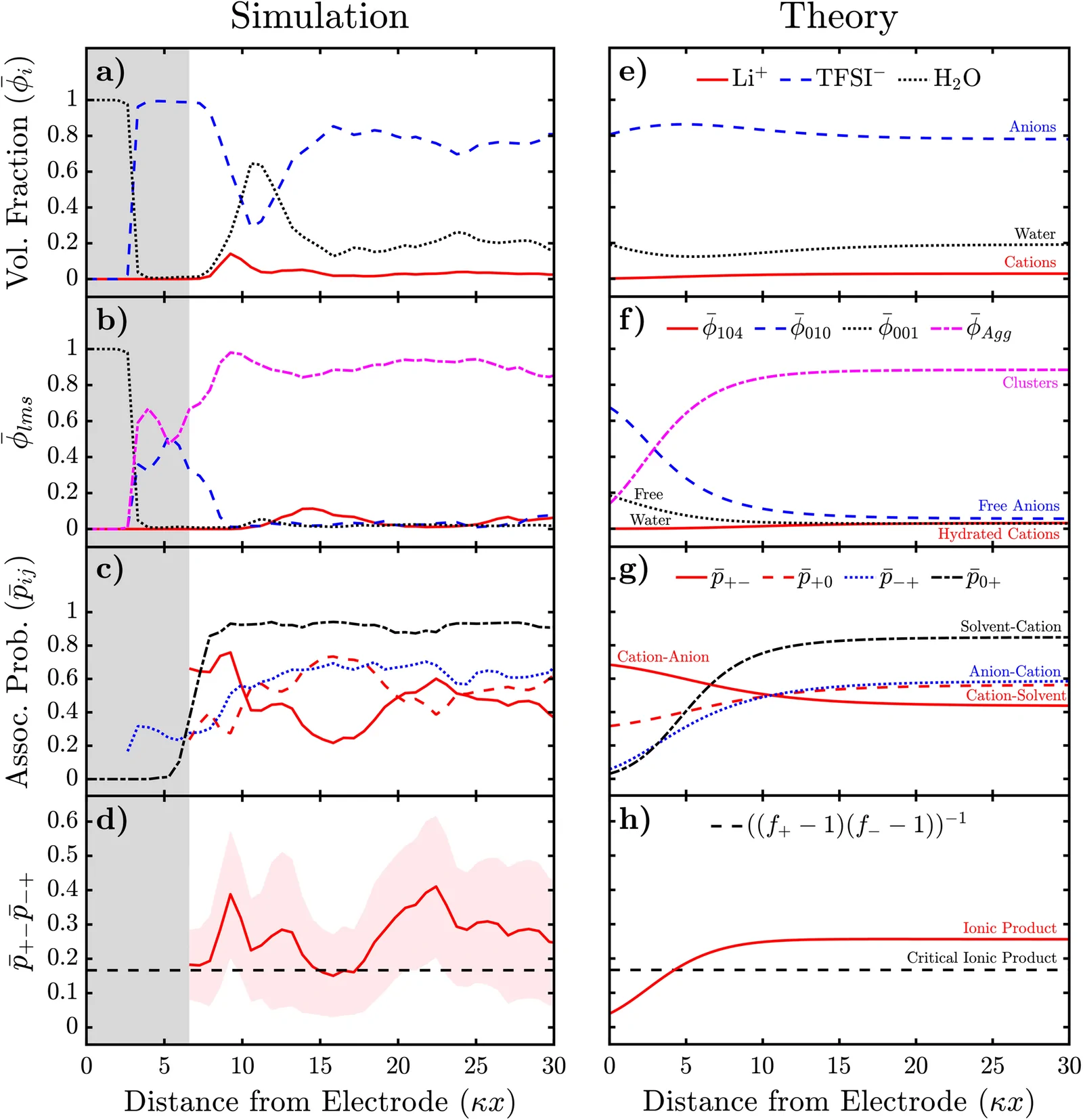
EDL of 21 m WiSEs at the positive electrode (*σ* = 0.2 C m^−2^) as a function from the interface, in dimensionless units, where *κ* is the inverse Debye length, from simulations (a–d) and theory (e–h). In the former, the grey region indicates the minimum distance from the electrode at which a species was never found. (a and e) Volume fraction of each species (*φ̄*_*i*_). (b and f) Volume fractions of hydrated cations (simulation *φ̄*_10*x*_ and theory *φ̄*_104_), free anions (*φ̄*_010_), free water (*φ̄*_001_), and aggregates and gel, if present (*φ̄*_Agg_). (c and g) Association probabilities (*p̄*_*ij*_). (d and h) Product of the ionic association probabilities, *p̄*_+−_*p̄*_−+_, where the dashed line indicates the critical line for gelation, with the shaded region denoting its standard deviation.

In Fig. 3, we compare the EDL predictions from MD simulations and theory for the positive (cathodic) electrodes of 21m LiTFSI WiSE. We display the changes of volume fractions in the EDL in Fig. 3(a) and (e), from MD and theory, respectively. The simulations predict *φ̄*_−_ to have a pronounced dip in the diffuse layer, before saturating just outside the Helmholtz region. Right at the interface, however, the anions are completely depleted and there is a substantial water layer. There is also a large accumulation of water associated with the dip in anion volume fraction in the diffuse EDL. Interestingly, there is a moderate increase in the Li^+^ volume fraction between the dip in *φ̄*_−_ and the peak in *φ̄*_0_ (at *x* ≈ 9*λ*_D_), before it is completely suppressed in the Helmholtz region. The theory predicts more modest changes in the EDL. Cations are slowly and monotonically suppressed towards the interface. In contrast, the anions are initially enriched relative to the bulk, before being depleted closer to the interface, reflecting the MD simulations qualitatively. Similarly, the water is initially depleted in the diffuse layer, but increases near the interface, showing the non-monotonic dependence observed in the simulations.

Further inspecting the common clusters and free species in the EDL reveals more insights into these changes in volume fraction, as shown in Fig. 3(b) and (f). In the MD, we observe a large increase in free anions, *φ̄*_010_, around the boundary of the Helmholtz region, which corresponds to the saturation of *φ̄*_−_ previously described. Similarly, the water layer right at the interface is solely free water. The hydrated cations do not accumulate in the positive EDL. However, we do observe a non-monotonic dependence of the aggregate/gel volume fraction as the electrode interface is approached. In the diffuse EDL, there is a large peak which corresponds well to the increase in Li^+^, indicating the cations are mainly bound up in aggregates. Moving towards the Helmholtz region, the aggregates/gel is suppressed, but they remain substantial in the middle of the Helmholtz region. The theory agrees reasonably well with these simulations, although the finer, non-monotonic details are missed. We find a strong suppression in hydrated cations and clusters/gel, and an accumulation of free anions and free water.

Next, we inspect the changes in association probabilities, as seen in Fig. 3(c) and (g). From the simulations, we find *p̄*_+0_ fluctuates in the bulk and diffuse region before being suppressed near the Helmholtz region. Similarly, we find *p̄*_0+_ is strongly suppressed to 0 in the Helmholtz region indicating that water prefers to be in the free state. These changes in association probabilities are captured qualitatively by the theory. We find *p̄*_+−_ also fluctuates in the bulk/diffuse EDL, before increasing slightly towards the Helmholtz layer, while the anion–cation association probability decreases in the EDL. The theory is able to qualitatively reproduce these changes in the ionic association probabilities.

In Fig. 3(d) and (h), we investigate where the percolating ionic network survives in the EDL. From the simulations, we find that in the bulk, the gel phase exists, but moving towards the diffuse and Helmholtz regions, we find large fluctuations where it is almost suppressed several times. Within the Helmholtz region no gel phase exists, since we know this is dominated by free water and free cations, with some clusters. We find the theory predicts a monotonically decreasing percolation probability, which just dips below the critical condition at the interface, indicating that the gel phase is destroyed there.

**Fig. 4:**
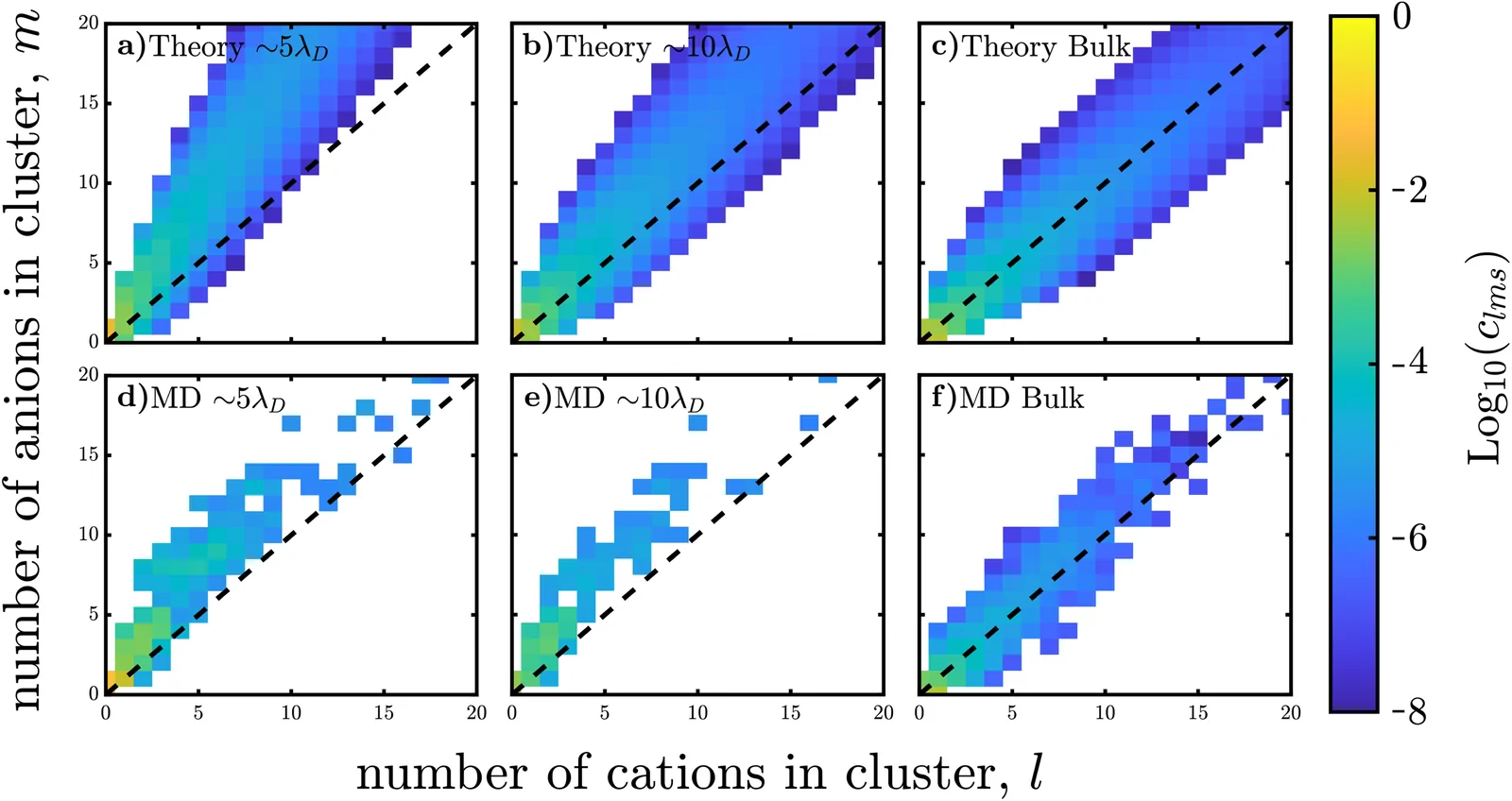
Cluster distribution of 21 m water-in-LiTFSI near the positive electrode (*σ* = 0.2 C m^−2^) at different distances from the interface, from theory (a–c) and simulations (d–f).

In Fig. 4, we further inspect the cluster distribution, from theory and simulation, at various positions in the EDL at positive electrodes. We will not recap the bulk as it was previously described. In the diffuse layer ∼10*λ*_D_ from the interface, the simulations predict a negatively biased cluster distribution. The simulations predict that the concentrations of the larger clusters increase relative to the bulk, but some of the larger aggregates are suppressed relative to the bulk. The theory predicts that the cluster distribution has a negative bias, with the presence of larger clusters than in the bulk. At ∼5*λ*_D_ similar observations are found from both theory and simulation.

### 4.3. Thermodynamic stability and interphase formation

Having further validated that our theory captures the trends in composition and coordination environments in the EDL of 21 m LiTFSI WiSE,^74^ we turn to using this theory to understand thermodynamic effects that influence stability and interphase formation of these electrolytes.^62,66,84^ We achieve this through predicting the changes in thermodynamic activity in the bulk and EDL, and changes in concentration within the EDL.^62,74,84^

We start by deriving expressions for the thermodynamic activity in the bulk with the sticky-cation approximation,^62^ based on our free-energy-functional formalism.^74^ Generally, the activity of a species, *a*_*i*_, can be expressed as

28 ln *a*_*i*_ = *β*(*µ*_*i*_ − *µ*^*θ*^_*i*_),

where *µ*_*i*_ is the chemical potential and *µ*^*θ*^_*i*_ is the reference state chemical potential. Using this definition, we have for the bulk the following sticky-cation approximation activities of each species (neglecting gel terms, as we did for the closure relations):29
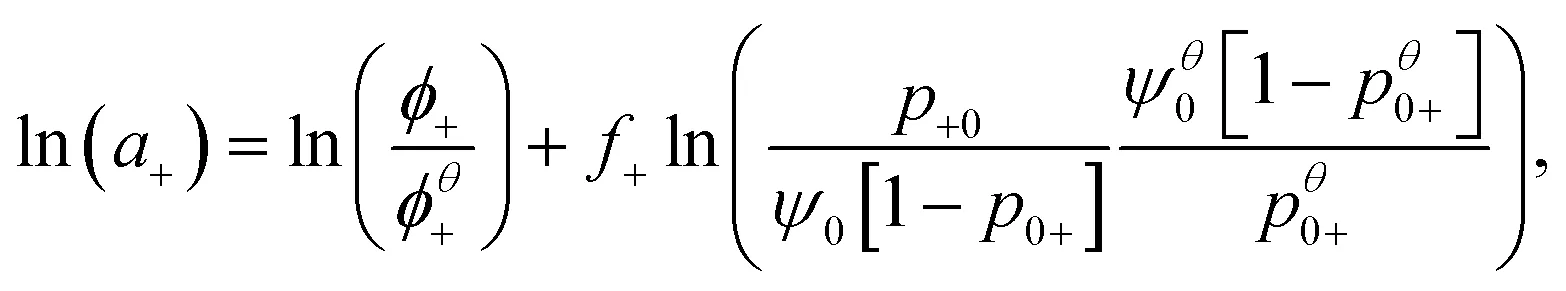
30
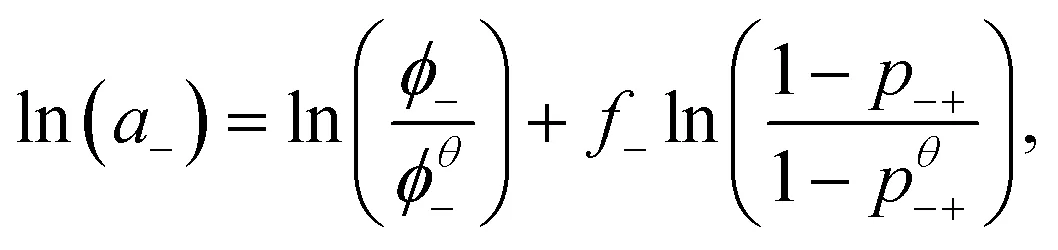
31
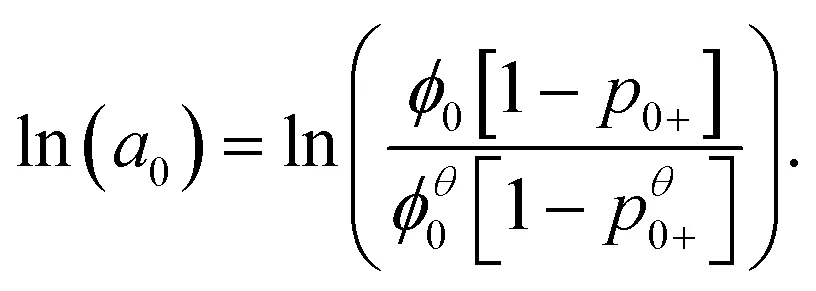
Note that, based on how we establish the chemical equilibrium, the chemical potential of cations (the same logic applies to anions and water) in an aggregate is equal to that of the free cation, and the free cation chemical potential does not depend on the cluster rank, so it is the convenient choice (*µ*^+^_*lms*_ = ∂*µ*_*lms*_/∂*l* = *µ*_100_ = *µ*_+_).^61,62^ In addition, we can also calculate the activity of certain species, such as the hydrated cation, as32
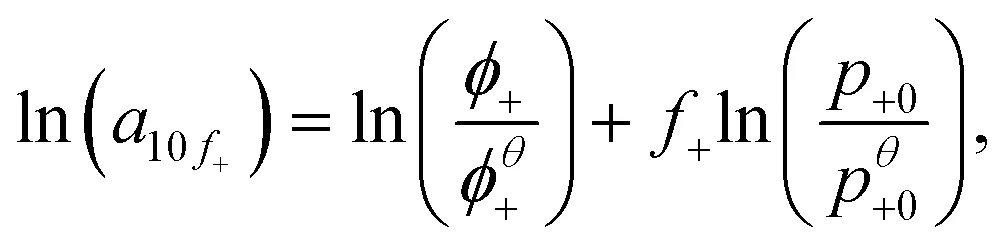
which is an important species in WiSEs. Note that in our theory the activities which represent the effective concentrations of species, are given by the concentrations of free species.^61^ This provides a simple and direct link, where all of the non-ideal changes are occurring through the associations.

In eqn (29)–(31), we take the reference state to be infinite dilution for the electrolyte. This corresponds to *ϕ*^*θ*^_0_ = 1, *p*^*θ*^_+−_ = *p*^*θ*^_−+_ = *p*^*θ*^_0+_ = 0, and *p*^*θ*^_+0_ = 1. We can use this reference state explicitly, but divergences remain. In practice, the standard is to take a small non-zero concentration of the electrolyte as the reference, which removes these divergences.^62^

**Fig. 5:**
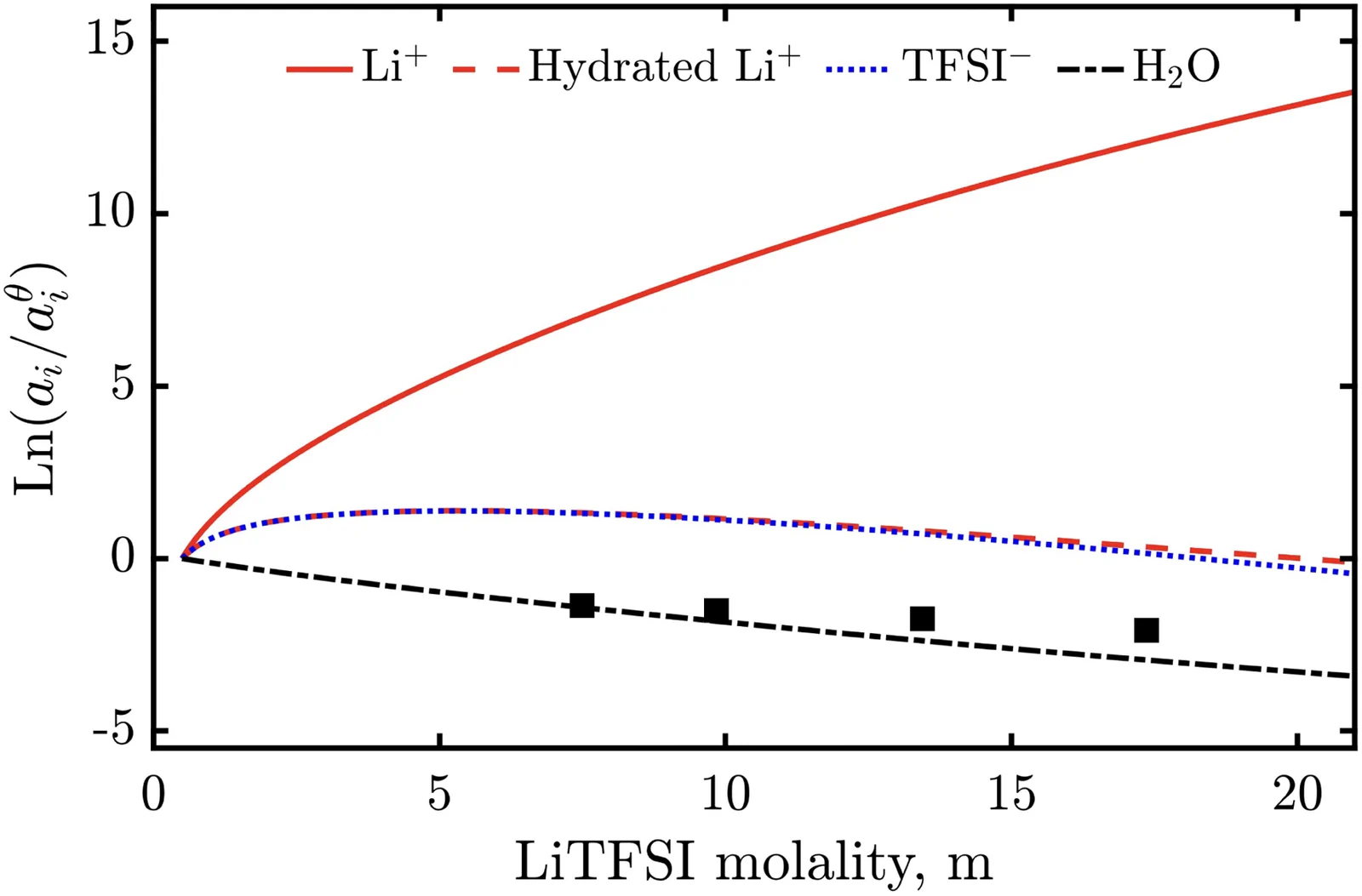
Activity of ions, water and hydrated cations in the bulk as a function of molality, computed with the sticky-cation approximation for LiTFSI. The reference concentration was taken to be 0.5 m. Experimental data (▪), reproduced and adapted from ref. 91, is referenced to align with the calculated activity at the lowest concentration.

In Fig. 5, we show the changes in activity as a function of salt concentration from these equations, for water, cations, anions and hydrated cations referenced against the 0.5 m state. Consistent with previous findings,^62^ we see that the water activity decreases with salt concentration, the anion activity initially increases with salt concentration before decreasing again at higher concentrations, and the Li^+^ activity monotonically increases with salt concentration.^62,91^ In addition, in Fig. 5 we show the activity of water derived from the experiments of Han *et al.*,^91^ where we have aligned the experiments with the theory predictions at the lowest concentration. In the experiments, the water activity also decreases with increasing salt concentration, but to a lesser extent than we predict with our theory. We find that the activity of hydrated cations tracks the activity of the anions.

**Fig. 6:**
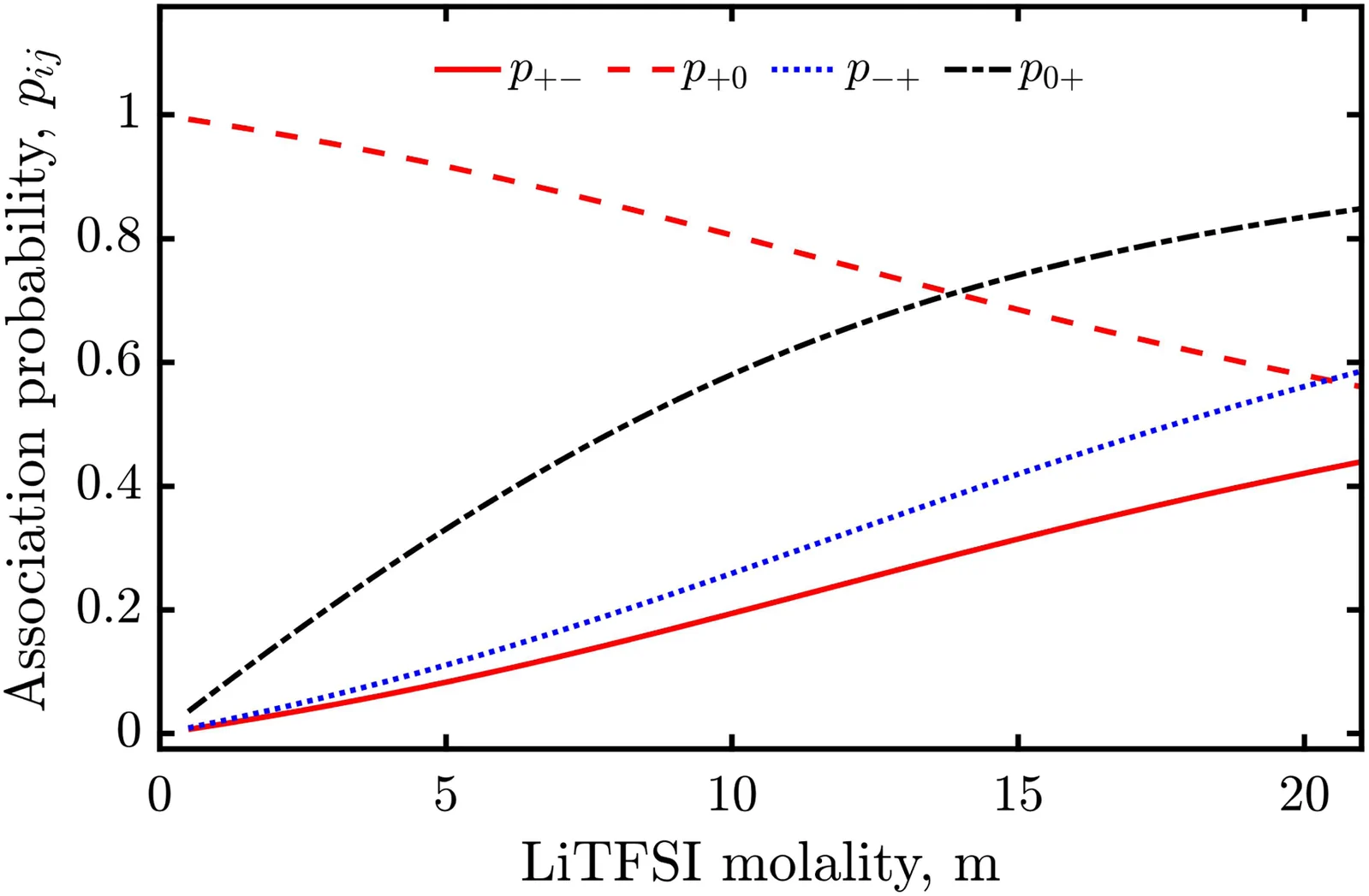
Association probabilities as a function of salt molality, from 21 m down to 0.5 m, computed with the sticky-cation approximation for LiTFSI.

To understand these changes in activity, all we need to know is how the association probabilities change with salt concentration, which provides the direct link of how activity depends on coordination environments as well as how the volume fractions of species change with salt concentration. In Fig. 6, we show how the association probabilities change with salt concentration. Using this information, we can determine that the anion activity is non-monotonic because the increase in *p*_−+_ dominates over the increase in *ϕ*_−_ at large salt concentrations. The cation activity monotonically increases because *p*_+0_/*ψ*_0_(1 − *p*_0+_) also increases with salt concentration. The activity of water decreases because of a decreasing *ϕ*_0_, but also because of an increasing *p*_0+_.

To understand why the hydrated cation activity follows that of the anion, we expand it in the smallness of *p*_−+_ and *p*_+−_ at low concentrations to give ln(*a*_10*f*+_) ≈ *f*_+_*p*_+−_, and ln(*a*_010_) ≈ *f*_−_*p*_−+_, which we can see must hold from the conservation of associations. According to hydration theory,^95^ the difference between the activity of cations and anions can be attributed solely to their different solvation environments. Our observation that the hydrated cation activity is close to the anion activity suggests that the hydration theory^95^ could be contained within, or be a limit of, our more general theory.

These changes in activity are a key part of the changes in thermodynamic stability of the electrolyte, and its ability to form a stable interphase. The changes in activity can be directly linked, through the Nernst equation, to changes in the redox potentials of reactions that can occur to form the interphases or to drive parasitic reactions.

Let us consider the SEI reaction from ref. 96, where the TFSI^−^ undergoes reduction and removal of fluoride, which combines with Li^+^ to form LiF:

33 Li^+^ + (R–F_3_)^−^ + e^−^(sec. electrons) ⇌ LiF + (R–F_2_)^−^,

where R = CF_3_–SO_2_–N–SO_2_–C. The change in the equilibrium reduction potential is given by the Nernst equation for this reaction:34
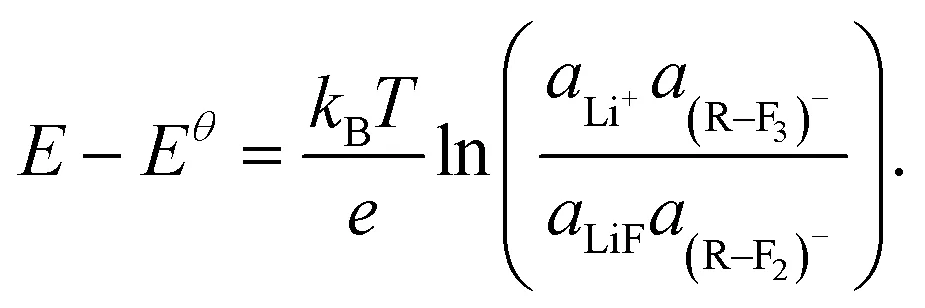
Taking the activity of the products to be ideal, *i.e.*, *a*_LiF_ = 1 and *a*_(R–F2)^−^_ = 1, the change in equilibrium redox potential is given by35
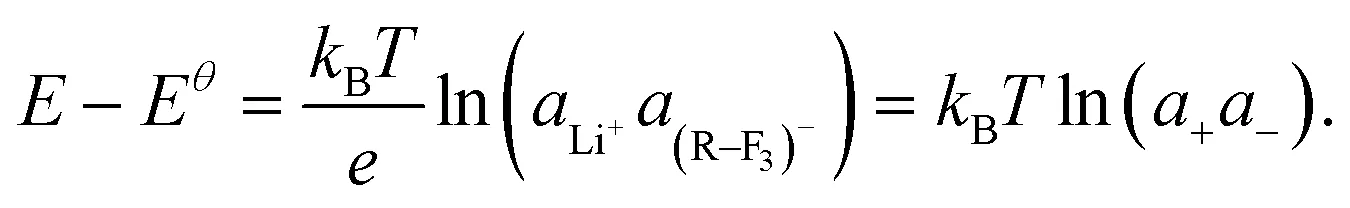
Therefore, increasing the salt concentration from 0.5 m to 21 m leads to a positive shift of approximately 0.35 eV in electrochemical reactions involving Li^+^, such as LiF formation in eqn (33). Similarly, other Li-containing salts formed in the SEI/CEI (Li_*x*_PO_*y*_F_*z*_, Li_2_O, Li_2_CO_3_, and LiOH),^97–99^ as well as their associated decomposition reactions,^100^ will undergo positive shifts in their electrochemical reduction potentials as the concentration increases from 0.5 m to 21 m due to changes in Li activity. Moreover, our theory shows that variations in Li^+^ activity play a significantly larger role in shifting these reduction potentials than changes in TFSI^−^ activity, the latter contributing a comparatively small negative shift of approximately −0.1 V over the same concentration range. These shifts lead to significant differences in the chemical composition of the interphases formed at 0.5 m and 21 m.

Next we consider the salt stabilization of water. At positive interfaces, suppression of the oxygen evolution reaction has been reported. Considering the oxidation of water, we have2H_2_O ⇌ O_2_ + 4H^+^ + 4e^−^.The Nernst equation for oxygen evolution reaction is36
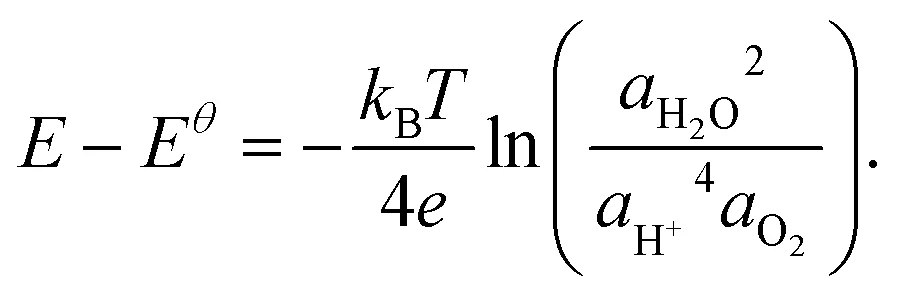
Assuming the activities of the products are ideal, *a*_H^+^_ ≈ 1 (*i.e.*, neglecting the role of pH) and *a*_O_2__ ≈ 1, we have37
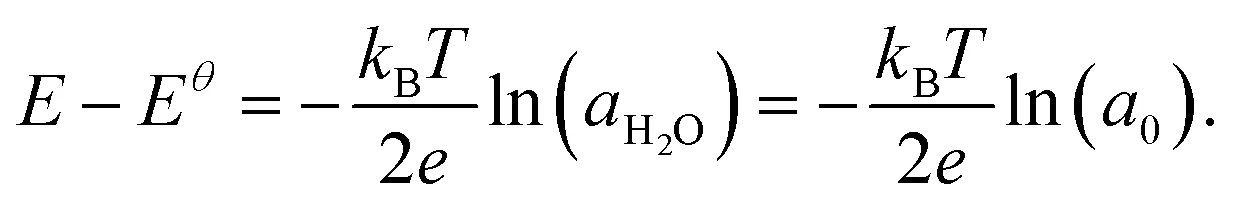
This states that changing the salt concentration from 0.5 m to 21 m shifts the oxidation potential of water by 5 mV. Therefore, it becomes slightly less thermodynamically favourable to oxidise water with increasing salt concentration. This indicates improvements in electrochemical stability for the water mainly arise from kinetic contributions as opposed to thermodynamic contributions at more positive potentials.

In a similar way, consider the reduction of water at negative potentials:2H_2_O + 2e^−^ ⇌ H_2_ + 2OH^−^.The Nernst equation for this reaction is38
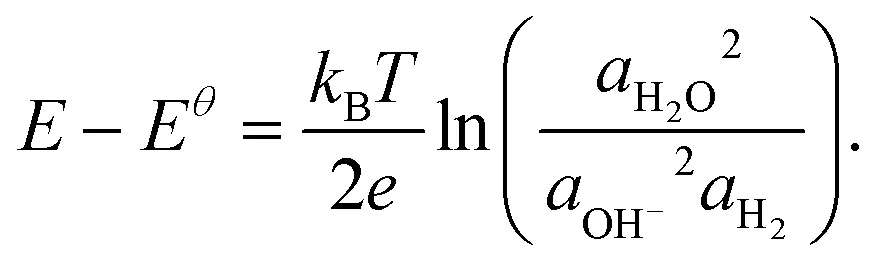
Again, assuming that the activities of the products are ideal, *a*_OH^−^_ ≈ 1 (*i.e.*, neglecting the role of pH) and *a*_O_2__ ≈ 1, we arrive at39
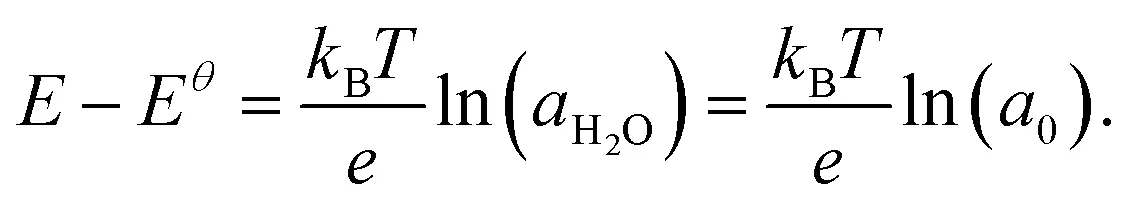
Increasing the salt concentration from 0.5 m to 21 m shifts the reduction potential of water by −10 mV, making water reduction slightly less thermodynamically favorable at higher salt concentrations. This shift contributes, in part, to the apparent expansion of the electrochemical stability window. These results demonstrate that increasing salt concentration appreciably alters SEI composition by promoting the formation of Li salts, while thermodynamically suppressing the reduction of water. While the thermodynamic stability at positive potentials is slightly increased, kinetic limitations of the OER likely dominate resulting in the improved oxidative stability observed in WiSEs.

Next, we look into the thermodynamic effects that can alter reaction kinetics at the interface.^70,101^ In expressions for the current densities, there is often a linear dependence on the concentration of the species reacting at the interface.^101^ The seminal example of which is Butler–Volmer reaction kinetics.^101^ Moreover, this intuitively makes sense, since what can react (and form the interphase or evolve gases) is what is at the interface between the electrode/interphase and the electrolyte, where the electron transfer processes can occur. Therefore, provided we are in a situation where transport limitations are not strong, such that an equilibrium theory can be used, looking at the concentration of key species in the EDL, especially right at the interface, can provide insight into changes in these redox reactions. For the reactions previously discussed, we consider how the concentration of reactants at the interface could influence the rates of these reactions.

For the formation of the inorganic SEI, we can inspect the surface concentrations of Li^+^ and TFSI^−^.^96^ As previously discussed, we found a large concentration of Li^+^ right at the interface of the negative electrodes, followed by a layer of water, and at further distances there is a significant concentration of TFSI^−^ anions. Further inspection showed that much of the Li^+^ and water were present in the form of hydrated Li^+^, but there was also a significant fraction of clusters. Since these clusters will involve Li^+^–TFSI^−^ at the interface, there should not be any kinetic reasons to limit the formation of LiF, and, in fact, these clusters should facilitate this reaction. At lower concentrations, as previously studied in ref. 74, the interface was significantly more populated with hydrated Li^+^ and there was an absence of clusters at the interface. Therefore, with increasing salt concentration, there is an increase in the thermodynamic stability of the electrolyte, but also the initial kinetics of the SEI formation should be more facile, which can also contribute to a longer-term kinetic passivation through the formation of this SEI.^96^ On the other hand, for the oxidation of water at +0.2 C m^−2^ interfaces, we find substantial water right at the interface.^96^ While this is a large surface charge, as studied in ref. 74 and 84, at lower surface charges there can be a depletion of water, which would suppress the kinetics of the oxygen evolution reaction.

Not only can we inspect the changes in concentration within the EDL, but we can also derive expressions for the activity within the EDL.^102,103^ Again, within the sticky-cation approximation, and neglecting the gel terms and the electrostatic contributions to the electrochemical potential, we arrive at40
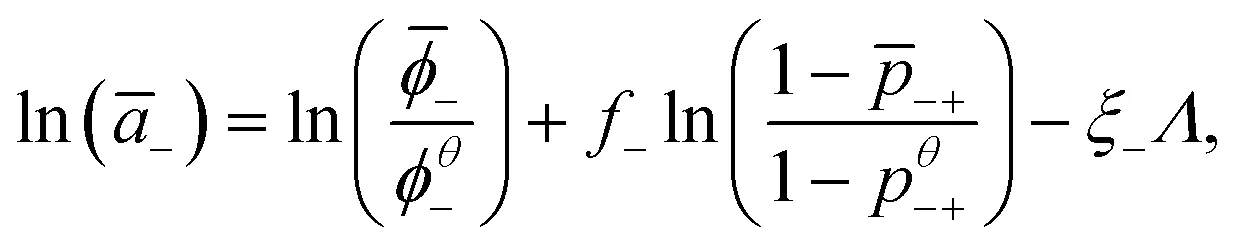
41
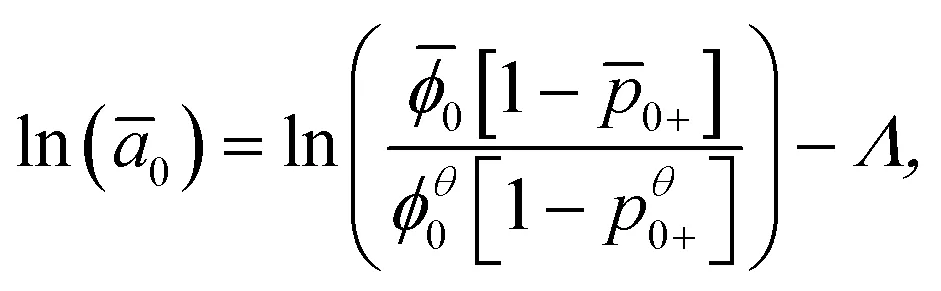
42
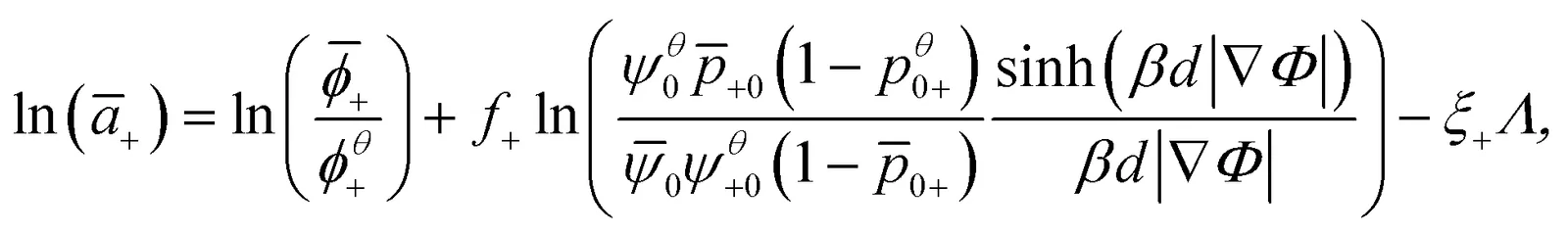
or equivalently,43
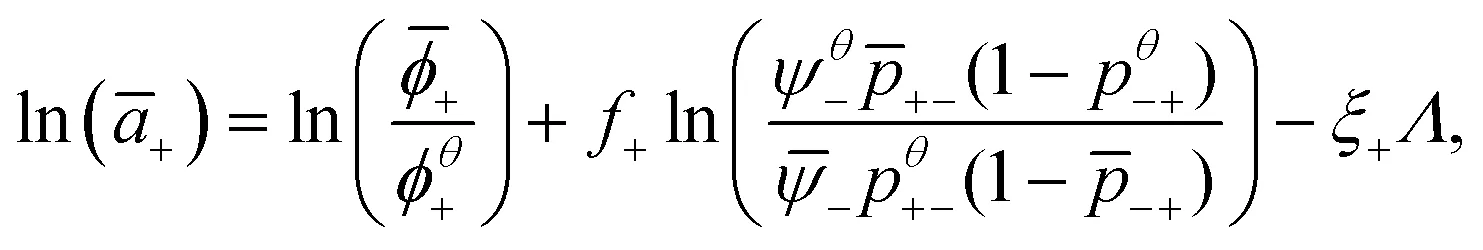
similar to the activity expressions previously stated.

**Fig. 7:**
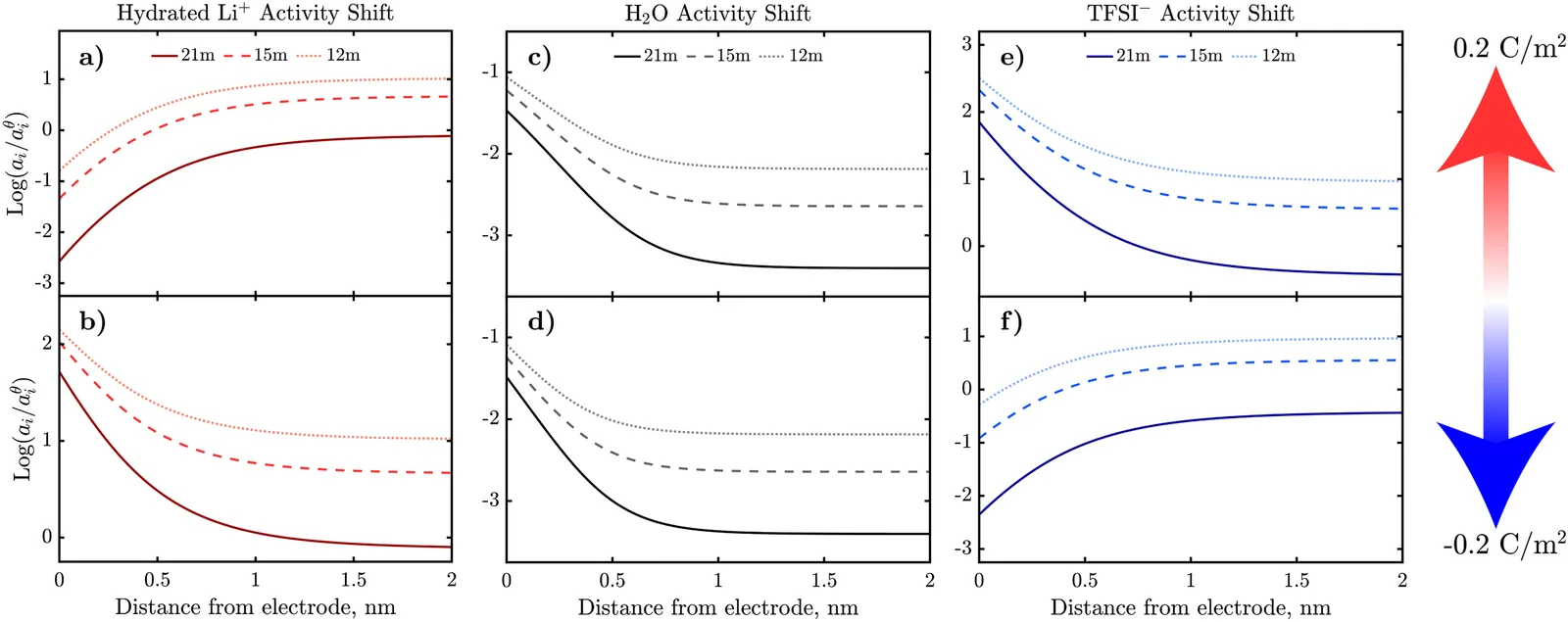
Activity of species within the EDL as a function of distance from the electrode, at various concentrations, at the indicated surface charges (a, c and e at 0.2 C m^−2^ and b, d and f at −0.2 C m^−2^) with a temperature of 300 K.

In Fig. 7, we display how the activity changes within the EDL for three concentrations, and for surface charges of ± 0.2 C m^−2^. As expected from the previously described EDL profiles in Fig. 2–4, the activity of cations increases at negative electrodes, but decreases towards positive electrodes. This change is more pronounced with increasing concentration. Analogously, the anion activity decreases in the EDL of negative electrodes, but increases in positive EDLs, with larger effects for more concentrated solutions. Whereas, we find that the activity of water increases for both polarizations of the electrode, since water is attracted by electric fields.^74^

While we can determine the activity of species in the EDL,^102^ which is commonly used terminology in the electrocatalysis community,^103^ we note that this can lead to some confusion. In the Nernst equation, the electrochemical equilibrium between the oxidised and reduced species is considered in the bulk.^62,66^ We also investigate the changes in composition in the EDL arising from establishing equilibrium with the bulk, which is conceptually similar to the Nernst equilibrium. However, the Nernst equation considers the changes in chemical potential of the species with composition, whereas in the EDL the composition changes due to the applied field, but the chemical potentials of the species are equal to their bulk values.^65,74^ Therefore, one must not think that the activity of species within the EDL can drastically change the redox potentials. The relevant quantities within the EDL are the concentrations of the species, but these quantities appear in the kinetic equations for these electron transfer processes, not for thermodynamic changes.

## 5. Discussion

Here, we presented a theory validated from MD simulations that predicts the structure of the EDL in 21 m LiTFSI WiSE,^74^ in addition to the thermodynamic activities of the species,^62^ which allows for statements about changes in stability and reactivity towards interphase formation of the electrolyte. Our theory is simple, analytical and has at most one free fitting parameter (*α*), with the handful of parameters it does have being easily obtained from MD simulations.^61^ Moreover, it directly links changes in coordination environments to these changes in activity, thereby confirming correlations between data that have previously been drawn.^78^ We believe our approach has advantages over others, such as the liquid-Madelung-potential ^60^ or OPAS,^62,66^ since only one MD simulation is required to parameterize the model, which can then make predictions across different compositions in the bulk and the EDL.

Not only that, but our approach can be readily extended to other electrolytes. For example, we have also studied ionic liquids,^63^ salt-in-ionic liquids^64,73^ and several conventional carbonate battery electrolytes.^66,76^ In terms of battery electrolytes, Phelan *et al.*^66^ found similar trends in the activities of salts and solvents. These predictions were validated using OPAS MD simulations, as well as by shifts in the Li^+^/Li redox potential observed experimentally.^66^ There, the liquid-Madelung potential was also compared,^60^ where much poorer agreement with OPAS MD simulations and experimental measurements was found.^66^

Therefore, our formalism can be applied to a wide variety of electrolytes of interest in energy-storage technologies. As described in the Theory section, the presented version of the theory can only strictly be applied to an electrolyte where two components have *f*_*i*_ ≥ 2; otherwise, it is not known how the aggregates are connected.^104^ However, despite this, the mass action laws, conservation of associations, and expressions for the free concentrations of ions are independent of this approximation, and can be applied more generally to more complicated electrolytes with more components.^66,104^ These equations are44
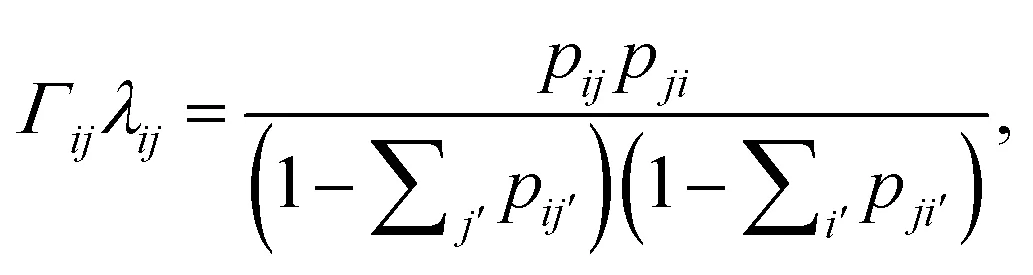

45 *Γ*_*ij*_ = *Ψ*_*i*_*p*_*ij*_ = *Ψ*_*j*_*p*_*ji*_,

46
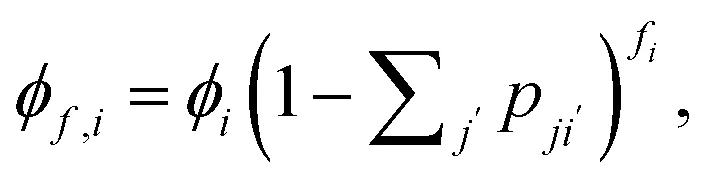
where *ϕ*_*f*,*i*_ has been used to denote the free volume fraction of species *i*. While these equations allow for less-restrictive functionalities to be used (any choice of functionality can be used), the electrolyte should still adhere to other assumptions of the theory (such as branched aggregates forming instead of crystalline phases, and an association being well described by one association constant/environment^105^), and needs to be well described by these approximations.

Moreover, assuming the species only interact through associations, we can use them in the activity expressions47
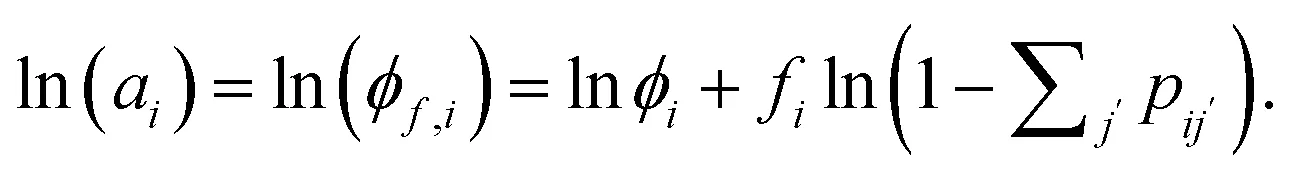
With this, we can discuss more generally the design principles of electrolytes for shifting the activities in desired directions. To increase the activity of a species *i*, we can increase the concentration of *i* or reduce *∑*_*j*′_*p*_*ij*′_. While we have direct control over the composition of the electrolyte, how the species interact and coordinate is determined more by their chemistry. For example, in the context of high-entropy electrolytes for Li-metal anodes, it was suggested that including more components of similar chemistry improves transport properties while maintaining the stability of the electrolyte.^106^ It is clear from eqn (47) and the discussion in ref. 76 that the salt activity of a high-entropy electrolyte would not be altered, which is consistent with experimental observations.^106^ With the generalised approach, we can also understand many-anion high-entropy electrolytes.^107^ Provided all anions interact with the cation with the same association constant, have the same functionality, and the overall volume fraction of anions is the same, there should again be no net effect on activity. Any changes in these variables could, however, alter the predictions of activity, but it is likely that these changes would be small (compared to the large changes over a wide range of salt concentrations). In those many-anion high-entropy electrolytes, similar SEI-forming abilities were reported, which we can corroborate with our approach. In addition, improved transport properties and operating temperatures were reported,^107^ but further investigation is required to comment on the design principles for transport properties.

Moreover, the EDL theory developed is built on the equilibrium of free species.^65^ Therefore, a general closure relation can be stated as

48 *φ̄*_*f,i*_ = *ϕ*_*f*,*i*_*F*(*Φ*,∇*Φ*,*α*),

where *F*(*Φ*,∇*Φ*,*α*) is the response of the species to electrostatic potentials and fields. Note that an equivalent set of mass-action laws and conservation of associations applies in the EDL.^65^ Therefore, the EDL can be investigated for more complicated electrolytes.

What this approach lacks, however, is the microscopic insight into changes in association constants with composition.^64^ This could, however, be overcome empirically, and this simpler set of equations might provide greater utility than the full complexity of the theory presented here.

In the context of interphase formation, we have several key thermodynamic insights that can be widely applied to other electrolyte systems. However, the role of the electrode in our theory, and also in the simulations, is somewhat lacking, other than it holding the charge in the EDL. This was identified in ref. 75, where specifically the Helmholtz-layer solvation environments of conventional battery electrolytes was studied. However, that approach was still mainly focused on the electrolyte properties, with the role of the electrode entering through a reduction in the apparent functionality of species (and a generic surface-interaction potential). Therefore, to gain more insight into the role of different electrodes, such as Li-metal *vs.* graphite, these surface interactions need to be included more explicitly.

While we have discussed thermodynamic effects from our simple model, which provides insight into interphase formation, it still cannot directly predict what is going to form in the SEI or CEI. The changes in activity correlate with changes in frontier orbitals,^66^ but this does not provide an explicit link to interphase formation. Therefore, while our approach can provide guiding principles, chemically accurate predictions of interphase formation require atomistic approaches. Such approaches include reaction networks,^108^ MLIPs, and adiabatic energy surfaces from DFT^109^ for coupled ion electron transfer (CIET) kinetics.^110,111^ While computationally challenging, these approaches can provide the chemical insight lacking in our approach.

Finally, beyond insights into electrolyte stability and interphase formation, the EDL predictions here are also of fundamental importance, as many open questions remain in the context of the EDL of highly concentrated electrolytes.^112,113^ Anomalous underscreening, the observation of extremely long force-decay lengths in surface force apparatus/balance (SFA/SFB) measurements, has puzzled the community for over a decade.^91,94,114–118^ Extensive ion pairing, network formation^114^ and “holes”^118^ acting as the charge carriers have been proposed to explain these measurements. Our theory accurately includes ionic aggregates and gels in a consistent EDL framework, but we still only predict screening lengths that are marginally longer than the Debye length. Therefore, our theory does not support the assumptions of these measurements, *i.e.*, that they arise from an equilibrium electrostatic effect. Recently, experimental evidence that these measurements are not at equilibrium has been reported.^119^ Further investigating these effects is important, with a particular focus on the non-equilibrium changes in associations in the EDL.^94^

Moreover, the close-range structure of the EDL of 21 m WiSEs, and other concentrated electrolytes, still remains to be fully understood.^120^ Our predictions, however, appear to match experimental findings remarkably well. Specifically, at negative electrodes, we predicted two distinct layers of Li^+^. Right at the interface, we predicted it to be dominated by hydrated cations, while the second layer contains some aggregates, before the bulk gel phase is reached. This is remarkably similar to the AFM and SFA measurements of Han *et al.*,^91^ where the first layer was found to be composed of hydrated Li^+^, and the second layer contained some ion pairs, before the aggregate size sharply increased, with nanostructure being detected as far out as 60 nm.

## 6. Conclusions

Overall, we extended our comparison between atomistic MD simulations and theory for the EDL in LiTFSI WiSE to 21 m. To achieve this from theory, we used the Boltzmann closure relations and introduced a charge-rescaling factor. Qualitatively, good agreement between theory and simulation was found. This theory was then used to predict thermodynamic effects that could influence the stability and reactivity towards interphase formation. We first computed the changes in activity in the bulk, where we found that these changes should result in a positive increase in the redox potentials for the formation of Li salts, such as LiF, in the SEI and thermodynamically suppress the hydrogen evolution reaction at negative potentials. The influence of the activity change on the oxygen evolution reaction was also explored. In addition, we used the equilibrium EDL concentrations to make predictions about how the kinetics of these reactions could be affected, where we found that the formation of aggregates in the EDL should facilitate the formation of Li salts in the SEI. We presented a discussion of how this formalism could be extended to more complicated electrolytes, and applied to a wide variety of energy-storage technologies.

## Conflicts of interest

The authors declare no conflicts of interest.

## Data Availability

Data and scripts for this paper are available at https://github.com/DMMarkiewitz/Faraday_Discussions_2026 and https://doi.org/10.5281/zenodo.20450228. This includes scripts to solve the Boltzmann closure relation, implicit Poisson solver for EDL quantities, MD analysis scripts, MD data, and scripts to produce the figures comparing simulations and theory.
